# The Effect of Ideological Identification on the Endorsement of Moral Values Depends on the Target Group

**DOI:** 10.1177/0146167218798822

**Published:** 2018-10-13

**Authors:** Jan G. Voelkel, Mark J. Brandt

**Affiliations:** 1 Tilburg University, The Netherlands

**Keywords:** morality, moral foundations, ideological identification, ideology, political psychology

## Abstract

Research suggests that liberals and conservatives use different moral foundations to reason about moral issues (moral divide hypothesis). An alternative prediction is that observed ideological differences in moral foundations are instead driven by ingroup-versus-outgroup categorizations of competing political groups (political group conflict hypothesis). In two preregistered experiments (total *N* = 958), using experimentally manipulated measures of moral foundations, we test strong versions of both hypotheses and find partial support for both. Supporting the moral divide hypothesis, conservatives endorsed the binding foundations more strongly than liberals even when a moderate target group was explicitly specified. Supporting the political group conflict hypothesis, both conservatives and liberals endorsed moral foundations more when moral acts targeted ingroup versus outgroup members. These results have implications for improving measures of moral values and judgments and point to ways to enhance the effectiveness of strategies aimed at building bridges between people from different political camps.

The moral judgments of liberals and conservatives conflict on many issues, whether equal rights for gays and lesbians, the levels of military spending, or the appropriate way to care for the nation’s poor. Some perspectives suggest that these different moral judgments arise from the different moral values that people who identify as liberals and conservatives endorse (e.g., Caprara, Schwartz, Capanna, Vecchione, & Barbaranelli, 2006; Graham, Haidt, & Nosek, 2009; Janoff-Bulman, 2009). This perspective makes sense when thinking of liberals and conservatives as endorsing different abstract value systems. Indeed, ideologies are often thought to be characterized, if not defined, by their connections with more abstract values (e.g., Jost, Glaser, Kruglanski, & Sulloway, 2003). However, recent work has found that liberal and conservative identification functions like other social identities and not like a more abstract ideology (Huddy, Mason, & Aarøe, 2015; Kinder & Kalmoe, 2017). From this perspective, conflicts between liberals and conservatives do not represent a conflict over competing moral values or even necessarily competing issues (Mason, 2018), but rather a conflict over opposing group identities. Here we test how these two perspectives contribute to ideological differences in moral foundations.

## The Moral Divide Hypothesis

Moral foundations theory (MFT; Haidt, 2012) predicts that moral conflicts are related to differences in the endorsement of at least five moral foundations which people use to form and inform their moral judgments. These moral foundations are thought to represent evolved psychological mechanisms that are common across cultures (Graham et al., 2011). However, the extent to which members of a culture endorse each of the moral foundations varies.

When applied to politics within a culture, liberals more strongly endorse the care/harm and fairness/cheating foundations (the so called “individualizing” foundations), whereas conservatives more strongly endorse the loyalty/betrayal, authority/subversion, and sanctity/degradation foundations (the so called “binding” foundations; Graham et al., 2009). In short, liberals and conservatives consider different moral foundations important and relevant when making moral judgments, which leads to ideological conflict in a number of domains (see also Feinberg & Willer, 2015; Voelkel & Feinberg, 2017).

## The Political Group Conflict Hypothesis

However, differences in moral foundations might be explained by political ingroup versus outgroup conflicts (cf. Frimer, Gaucher, & Schaefer, 2014). The political group conflict hypothesis suggests that ideological differences in moral foundations are not necessarily due to differences in moral values per se but are driven by ingroup-versus-outgroup categorizations. This hypothesis has its roots in research that emphasizes that people’s thoughts, attitudes, and behaviors are strongly influenced by the ideological groups they identify with (e.g., Brandt, Reyna, Chambers, Crawford, & Wetherell, 2014) and is consistent with work suggesting that people’s ideological identifications function like a group identification (e.g., Huddy et al., 2015; Kinder & Kalmoe, 2017). According to this view, liberals and conservatives may selectively and flexibly endorse moral values depending on the target group of the moral act.

For example, the typical finding that conservatives endorse the moral value of respect for authorities could be an artifact of the tendency for participants to assume that authorities are conservative. When responding to the value of obeying *liberal* authorities, it appears that liberals are more likely to endorse the moral value of authority than are conservatives (Frimer et al., 2014). This suggests that conservatives support authority not because they are generally more concerned with authority, but because authorities are associated with a conservative ideology. What appears to be a conflict over moral foundations instead is a political group conflict.

A similar argument can be made for the fairness foundation. The strong version of the moral divide account predicts that liberals should be more likely to endorse the fairness foundation no matter the target group. The political group conflict account makes a different prediction: Liberals will condemn unfair treatment of liberal groups and groups stereotyped as liberal more than conservatives. However, conservatives will condemn unfair treatment of conservative groups and groups stereotyped as conservative more than liberals. Such a finding would suggest that the fairness foundation is not unique to liberals, as both groups care about fairness *for their own political ingroups*. The political group conflict hypothesis predicts that moral acts are evaluated based on whether the actor and the target belongs to or are associated with political ingroups or outgroups.

## The Moral Foundations Questionnaire Cannot Test These Two Perspectives

The political group conflict hypothesis is in the position to explain much of the research that is cited as support for the moral divide hypothesis without including the assumption of different moral foundations stemming from evolved modules for liberals and conservatives (cf. Haidt, 2012; Haidt & Joseph, 2004). This is because the items that are used to measure moral foundations are partly confounded by the target groups that are mentioned in the moral foundation items. For instance, the item “Whether or not someone’s action showed love for his or her country” from the Moral Foundation Questionnaire (MFQ; Graham et al., 2011) is designed to measure the endorsement of the loyalty/betrayal foundation. However, a person’s lack of loyalty toward one’s country does not imply that loyalty is not an important value for them. As conservatism is associated with blind patriotism (Schatz, Staub, & Lavine, 1999), this item may contribute to the observed ideological differences in the loyalty/betrayal foundation score due to differences in the evaluation of the target group (i.e., the country), but not necessarily due to the overall importance of loyalty. Specifying a different target group (e.g., “Whether or not someone’s action showed love for his or her labor union”) might find a liberal advantage in loyalty. This is just one example from one MFQ item, but a similar critique can be leveled at several of the MFQ items (see Table SM1 in the supplementary materials).

A second problem is that when groups are given ostensibly neutral names (e.g., “authorities”), the neutral names may still be contaminated by ideological stereotypes. For instance, asking participants to name authorities resulted in a list dominated by authorities associated with conservative political agendas (Frimer et al., 2014). When the MFQ asks people to indicate how important it is to show respect for authorities, participants may report how important it is to show respect for *conservative* authorities even if no conservative authority is mentioned. This is a problem beyond the authority domain. For example, the questions tapping into the fairness foundation refer to rights and discrimination and this is likely interpreted as discrimination of groups who are liberal or emblematic of liberal causes. If it were changed to specify discrimination of conservatives, it would likely shift the association between ideological identification and the endorsement of the fairness foundation (cf. Brandt et al., 2014).

In short, the content of the MFQ confounds general moral foundations and people’s stereotypes and reactions toward specific target groups and ideological ideas. Therefore, the MFQ cannot tease apart the moral divide and the political group conflict hypotheses. There is some research explicitly supporting the political group conflict hypothesis for the authority foundation (Frimer et al., 2014). While MFT claims that liberals endorse the authority foundation to a lesser extent than conservatives, the ideological identification–authority relationship is reversed when the authorities are associated with an explicitly liberal ideology (Frimer et al., 2014). A less direct study (Frimer, Tell, & Motyl, 2017) identified the legality of the Keystone XL oil pipeline as a policy on which liberals aim to protect the sanctity of the earth (see also Frimer, Tell, & Haidt, 2015) while conservatives aim to promote fairness. This suggests that, for some issues, liberals may be more sensitive to violations of moral sanctity and purity than conservatives just as conservatives may be to violations of fairness and justice.

Based on these results, researchers have developed alternative measures of moral foundations and judgments. For instance, the moral foundations vignettes (MFVs) explicitly avoid “overtly political content and reference to particular social groups” (Clifford, Iyengar, Cabeza, & Sinnott-Armstrong, 2015, p. 1181). Consistent with this goal, ideological identification correlates with moral foundations measured with the MFV (except for the sanctity/degradation foundation) at weaker levels than it does when measured with the MFQ (Clifford et al., 2015, Table 5). This is indirect evidence for the political group conflict hypothesis. Looking beyond morality measures, prejudice research has identified similar patterns: Both liberals and conservatives are prejudiced toward social groups characterized by a worldview that conflicts with their own (e.g., Brandt, 2017).

## The Current Research

Here we report two preregistered studies that test both the moral divide hypothesis and the political group conflict hypothesis across the five moral foundations proposed by MFT (Graham et al., 2009). Study 1 tests whether liberals’ and conservatives’ endorsement of moral values depend on the target groups by experimentally altering the political groups referenced in the MFQ (Graham et al., 2011), the most widely used measure of the endorsement of moral foundations. Study 2 conceptually replicates the first study by testing whether the moral judgments of behaviors related to the five moral domains (MFV; Clifford et al., 2015) depend on the ideology associated with the actor or target in the corresponding acts. Together, these two studies provide evidence that helps quantify the extent to which ideological differences in the endorsement of moral values are genuine, a result of ingroup versus outgroup thinking, or both.

In Study 1, we used a liberal target condition, a conservative target condition, and a moderate target condition compared with the original MFQ. For the liberal, conservative, and moderate target conditions, we used “liberals,” “conservatives,” and “middle-class people” as target groups respectively.^1^ In Study 2, we only used a liberal target condition and a conservative target condition compared with the original MFV.

We present the predictions that are derived from strong versions of the moral divide hypothesis and the political group conflict hypothesis. We use these strong versions as “temporary givens” to identify the differences between the two accounts (Zanna, 2004). The strong version of the *moral divide hypothesis* predicts that the relationship between ideological identification (higher scores are associated with more conservative identification compared with liberal identification) and moral foundations is not moderated by the target groups and is consistently negative and medium in size for the individualizing foundations and positive and medium in size for the binding foundations. In contrast, the *political group conflict hypothesis* predicts that the relationships between ideological identification and moral foundations are moderated by the target groups and that the degree of the differences (measured by the Ideological Identification × Condition interaction effects) is expected to be proportionate to the ideological distance between the targets. For the individualizing foundations, the political group conflict account predicts that the effect of ideological identification is negative and strong for a liberal target, small and not reliably positive or negative for a moderate target, and positive and strong for a conservative target. Contrary, for the binding foundations, the political group conflict account predicts that the effect of ideological identification is positive and strong for a conservative target, small and not reliably positive or negative for a moderate target, and negative and strong for a liberal target.

The goal of the current study is to test the strong versions of the moral divide hypothesis and the political group conflict hypothesis against each other. Although strictly speaking, the two accounts make different predictions in all but the original MFQ condition, some comparisons are particularly well suited to test the predictive power of the two hypotheses. Identifying the conditions that best distinguish the two hypotheses allows us to make strong inferences (Platt, 1964) about the role of moral divide and political group conflict. Such findings will help clarify to what extent the psychological processes invoked by each hypothesis are responsible for the observed data thus far. The following conditions best distinguish the two hypotheses:

1. In the moderate target condition, the political group conflict hypothesis predicts nonsignificant effects of ideological identification, whereas the moral divide hypothesis predicts significant negative effects of ideological identification for the individualizing foundations and significant positive effects of ideological identification for the binding foundations.
2. In the liberal target condition, the moral divide hypothesis predicts significant positive effects of ideological identification for the binding foundations, whereas the political group conflict hypothesis predicts significant negative effects of ideological identification for the binding foundations.
3. In the conservative target condition, the moral divide hypothesis predicts significant negative effects of ideological identification for the individualizing foundations, whereas the political group conflict hypothesis predicts significant positive effects of ideological identification for the individualizing foundations.

Although we present the two hypotheses in their strongest possible terms, they are not mutually exclusive. Much research has focused on value-related differences between liberals and conservatives (Caprara et al., 2006; Graham et al., 2009; Janoff-Bulman, 2009; Jost et al., 2003; Thorisdottir, Jost, Liviatan, & Shrout, 2007), but this research does not rule out that other factors influence the endorsement of moral values. Similarly, it is possible that political group conflict predictions help explain the data and that there are still general tendencies of liberals and conservatives to adopt certain moral values more strongly than others (e.g., Wetherell, Brandt, & Reyna, 2013). In short, support for both hypotheses is possible.

An example of a result consistent with such a complimentary perspective would be null effects of ideological identification on the three binding foundations in the liberal but positive effects in the other target conditions. This could indicate that both processes are at work, but push in opposite directions: First, conservatives endorse the binding foundations more strongly than liberals across all target groups. Second, liberals endorse all moral foundations more strongly than conservatives if the target group of moral acts consists of liberals. If both processes are of similar strength, this would result in null effects. Note that we did not preregister any complimentary hypotheses.

## Study 1

We test the two hypotheses by randomly assigning participants to one of four versions of the MFQ. These versions differed in whether the items were the original MFQ items or adapted to include a liberal, conservative, or moderate target group. The preregistration (including hypotheses and analysis plan), the materials, the data, and the analysis script for this study can be found here: https://osf.io/gghh7/ (preregistration) and https://osf.io/9tx7x/ (data, analysis script, and description of minor deviations from the preregistration).

### Method

#### Sample

Participants were Americans recruited from Amazon Mechanical Turk and received a small amount of money for their participation. A power analysis with G*Power (Faul, Erdfelder, Lang, & Buchner, 2007) showed that to have 80% power to detect the difference between a correlation of .34 in one condition and a correlation of 0 in another we need 130 participants per condition (520 participants in total).^2^ To account for potential missing data, we aimed to collect data from 550 participants.

Our initial sample size consisted of 582 cases. Participants were removed if they (a) had the same IP address (keeping only the first case of each IP address, *n* = 11 cases removed) or (b) had at least three missing values for one of the moral foundation dimensions or did not indicate their ideological identification (*n* = 29 cases removed). The number of participants excluded due to missing values did not differ significantly across conditions, χ^2^(3) = 2.15, *p* = .542. The final sample consisted of 542 participants (59% male, 41% female, *M*_age_ = 35.33, *SD*_age_ = 11.41; liberal target *n* = 135, conservative target *n* = 139, moderate target *n* = 130, original condition *n* = 138).

#### Design, procedure, and materials

Participants were randomly assigned to one of the four conditions. In the original condition, participants responded to the original MFQ^3^ (Graham et al., 2011). In the liberal, conservative, and moderate conditions, the structure and items remained the same, but liberals, conservatives, or middle-class people were added as targets for all items. For instance, one original MFQ item is, “Justice is the most important requirement for a society.” We adapted it to “Justice for people who are [liberal/conservative/from the middle class] is an important requirement for a society.” Participants responded to 30 items (six per foundation) on the same response scales as those of the original MFQ.^4^ The items for each moral foundation were averaged and formed reliable scales in all conditions (care/harm α range [.69, .81]; fairness/cheating α range [.70, .87]; loyalty/betrayal α range [.76, .83]; authority/subversion α range [.79, .84]; sanctity/degradation α range [.83, .89]). Participants also completed a short demographics survey, which included their identification with a political ideology on a scale from 1 (*very conservative*) to 7 (*very liberal*). We recoded the item to range from −3 to 3 with higher values indicating a more conservative ideology. Our sample leaned liberal (*M* = −0.38, *SD* = 1.83). There was no evidence that the average ideological identification differed in the four conditions (all *p*s > .161).

### Results

#### Confirmatory analyses

**Figure 1.**
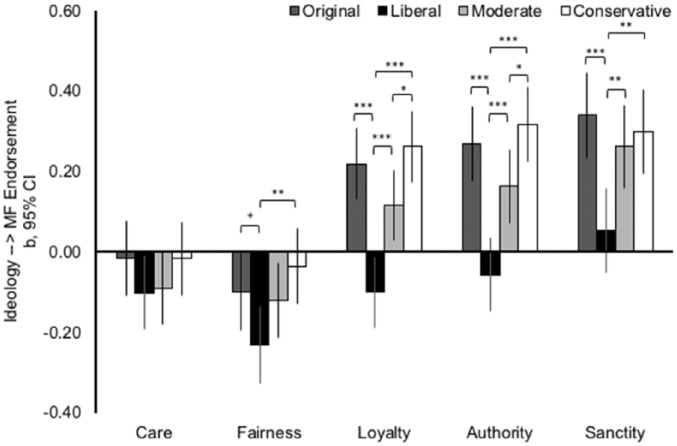
The unstandardized regression coefficients and 95% CIs for the effects of ideological identification in the four experimental conditions on endorsement of the five moral foundation in Study 1. *Note.* Significant differences between the slopes are computed using multiple regression analyses (see main text and supplemental materials). CI = confidence intervals. ^†^*p* < .10. *p < .05. **p < .01. ***p < .001.

To test our hypotheses, we conducted separate multiple regression analyses for each of the five different moral foundations. We included participants’ ideological identification, the experimental factors (three dummy variables), and the Ideological Identification × Condition interaction effects as predictors. We conducted four analyses per foundation that differed only in the reference category for the experimental factors to allow us to test all pairwise comparisons between the conditions. This allowed us to (a) compare the effect of ideological identification in the different conditions (six comparisons in form of interaction effects) and (b) estimate the effect of ideological identification in each condition (i.e., the effect of ideological identification when the condition is the reference category). The slopes of ideological identification as a predictor of moral foundations are plotted in Figure 1. Means and standard deviations, as well as full results of the multiple regression analyses underlying Figure 1 are provided in the supplementary materials (Tables SM3 & SM2.1-SM2.5).

##### Individualizing care/harm foundation

The effect of ideological identification did not differ across target group conditions, all *p*s for the six interaction effects >.201. As the interactions were not significant, we removed the interaction terms from the model and reestimated the model. Here we find that there is a small, negative effect of ideological identification, *b* = −0.06, *SE* = 0.02, *t*(537) = −2.43, *p* = .016, suggesting that liberals endorse the individualizing care/harm foundation more than conservatives across the four conditions.

##### Individualizing fairness/cheating foundation

We found a significant Ideological Identification × Condition interaction effect: the relationship between ideological identification and endorsement of the individualizing fairness foundation was significantly more negative in the liberal target condition than in the conservative target condition, interaction *b* = −0.20, *SE* = 0.07, *t*(534) = −2.85, *p* = .004. The relationship between ideological identification and endorsement of the individualizing fairness foundation was also marginally significantly more negative in the liberal target condition than in the original condition, interaction *b* = −0.13, *SE* = 0.07, *t*(534) = 1.91, *p* = .056. None of the other four ideology-target-group-condition interaction effects were significant, all *p*s > .103. The simple effect of ideological identification was negative and significant in the liberal target condition, *b* = −0.23, *SE* = 0.05, *t*(534) = −4.78, *p* < .001; in the moderate target condition, *b* = −0.12, *SE* = 0.05, *t*(534) = −2.53, *p* = .012; and in the original condition, *b* = −0.10,*SE* = 0.05, *t*(534) = −2.06, *p* = .039. The effect was not significant in the conservative target condition, *b* = −0.04, *SE* = 0.05, *t*(534) = −0.74, *p* = .461.

##### Binding loyalty/betrayal foundation

We found four significant Ideological Identification × Condition interaction effects. The relationship between ideological identification and endorsement of the binding loyalty foundation was significantly more negative in the liberal target condition than in the three other conditions, interaction *b*s < –0.21, *SE*s = 0.06, *t*(534)s < –3.45, *p*s < .001. The relationship between ideological identification and endorsement of the binding loyalty foundation was also significantly more negative in the moderate target condition than in the conservative target condition, interaction *b* = −0.14, *SE* = 0.06, *t*(534) = −2.29, *p* = .022. The other two Ideological Identification × Condition interaction effects were not significant, both *p*s > .106. The effect of ideological identification was positive and significant in the conservative target condition, *b* = 0.26, *SE* = 0.04, *t*(534) = 5.81, *p* < .001; in the original condition, *b* = 0.22, *SE* = 0.05, *t*(534) = 4.85, *p* < .001; and in the moderate target condition, *b* = 0.12, *SE* = 0.04, *t*(534) = 2.64, *p* = .009. In contrast, the effect was negative and significant in the liberal target condition, *b* = −0.10, *SE* = 0.04, *t*(534) = −2.25, *p* = .025.

##### Binding authority/subversion foundation

We found four significant Ideological Identification × Condition interaction effects. The relationship between ideological identification and endorsement of the binding authority foundation was significantly more negative in the liberal target condition than in the three other conditions, interaction *b*s < –0.21, *SE*s = 0.07, *t*(534)s < –3.31, *p*s < .001. The relationship between ideological identification and endorsement of the binding authority foundation was also significantly more negative in the moderate target condition than in the conservative condition, interaction *b* = −0.15, *SE* = 0.07, *t*(534) = −2.29, *p* = .022. The other two ideology-target-group-condition interaction effects were not significant, both *p*s > .109. The effect of ideological identification was positive and significant in the conservative target condition, *b* = 0.32, *SE* = 0.05, *t*(534) = 6.66, *p* < .001; in the original condition, *b* = 0.27, *SE* = 0.05, *t*(534) = 5.69, *p* < .001; and in the moderate target condition, *b* = 0.16, *SE* = 0.05, *t*(534) = 3.51, *p* < .001. In contrast, the effect was negative, but nonsignificant in the liberal target condition, *b* = −0.06,*SE* = 0.05, *t*(534) = −1.20, *p* = .231.

##### Binding sanctity/degradation foundation

We found three significant Ideological Identification × Condition interaction effects. The relationship between ideological identification and endorsement of the binding sanctity foundation was significantly more negative in the liberal target condition than in the three other conditions, interaction *b*s < –0.20, *SE*s = 0.08, *t*(534)s < –2.76, *p*s < .006. The other three ideology-target-group-condition interaction effects were not significant, all *p*s > .302. The effect of ideological identification was positive and significant in the original condition, *b* = 0.34, *SE* = 0.05, *t*(534) = 6.31, *p* < .001; in the conservative target condition, *b* = 0.30, *SE* = 0.05, *t*(534) = 5.55, *p* < .001; and in the moderate target condition, *b* = 0.26, *SE* = 0.05, *t*(534) = 4.95, *p* < .001. In contrast, the effect was substantially weaker and nonsignificant in the liberal target condition, *b* = 0.05, *SE* = 0.05, *t*(534) = 0.99, *p* = .324.

#### Exploratory analyses

We also inspected the main effects of the target conditions. The most consistent result was that, with regard to all five moral foundations, we found that participants in the original condition generally scored higher on the dependent variables than participants in the three other conditions (for full results see supplemental materials). Robustness checks suggest that neither including gender and age as control variables nor testing for nonlinear effects of ideological identification significantly affect our conclusions (for full results see supplemental materials).

### Discussion

The moral divide and political group conflict hypotheses make different predictions for several effects. The political group conflict hypothesis, but not the moral divide hypothesis, predicts significant Ideological Identification × Condition interaction effects: Across all of the moral foundations, except for the care/harm foundation, there is at least one significant Ideological Identification × Condition interaction effect in the predicted direction, indicating that the target group affects people’s endorsement of the moral foundations. However, not all of the interaction effects predicted by the group conflict hypothesis were confirmed. Here we assess how well each of the hypotheses does when making the predictions that best distinguish the two hypotheses.

1. Effects of ideological identification in the *moderate target* condition: The political group conflict hypothesis predicts nonsignificant effects of ideological identification across all five foundations, whereas the moral divide hypothesis predicts significant negative effects of ideological identification for the individualizing foundations and significant positive effects of ideological identification for the binding foundations. The data primarily support the moral divide hypothesis.
2. Effects of ideological identification on endorsement of the binding foundations in the *liberal target* condition: The moral divide hypothesis predicts significant positive effects of ideological identification, whereas the political group conflict hypothesis predicts significant negative effects of ideological identification. Support for the political group conflict hypothesis was only found for the loyalty foundation, where there was a significant negative association between ideological identification and loyalty endorsement. This does not mean that the effects are consistent with the moral divide hypothesis as the association between ideological identification and endorsement of the other two binding foundation in the liberal condition was not significantly different from zero.
3. Effects of ideological identification on endorsement of the individualizing foundations in the *conservative target* condition: The moral divide hypothesis predicts significant negative effects of ideological identification, whereas the political group conflict hypothesis predicts significant positive effects of ideological identification. In this case, neither perspective received support. The associations between ideological identification and endorsement of the individualizing foundations in the conservative target group condition were not significantly different from zero.

The results of Study 1 draw a more nuanced picture than provided by either of the two accounts alone. Consistent with the political group conflict hypothesis, we found that the effect of ideological identification depended on whether moral acts involved liberal or conservative groups. Consistent with the moral divide hypothesis, we found the pattern identified by MFT (liberals score higher on the individualizing foundations and conservatives score higher on the binding foundations) in the moderate target condition. This suggests that both differences in the endorsement of certain moral values and ingroup–outgroup thinking help explain the importance accorded by liberals and conservatives to different moral foundations. We come back to this in the “General Discussion” section.

## Study 2

In Study 2, we aimed to replicate our results of Study 1 with a different assessment of moral foundations—the MFV (Clifford et al., 2015). These vignettes were developed to represent violations of a particular moral foundation while controlling for other factors, such as syntactic structure, complexity of the vignettes, and, most importantly for our purposes, references to particular social groups (Clifford et al., 2015). Therefore, we removed the moderate target condition resulting in a three condition between-subjects design: the liberal target condition, the conservative target condition, and the original condition. In addition, some of the items in Study 1 may have appeared odd to participants because of the necessary changes to create the experimental conditions. For example, it is possible the transformed items of the loyalty dimension measured liking of groups instead of the endorsement of loyalty values. In Study 2, the structure and number of vignettes allowed us to more easily select vignettes so that the items read naturally after the specification of a target group.

The predictions of the moral divide hypothesis and the political group conflict hypothesis are equivalent to Study 1. With regard to the original condition, we believe that a strong version of the political group conflict hypothesis would suggest that the effect is expected to be small and not reliably positive or negative because the MFVs avoid reference to social groups (Clifford et al., 2015). However, it is also known that people infer ideological information from otherwise neutral stimuli (Frimer et al., 2014) and ideological differences on some dimensions have been found using the original versions of these stimuli (Clifford et al., 2015). Therefore, whereas the avoidance of references to social groups should weaken the effects, we expect to replicate the pattern of results expected by the moral divide hypothesis in the original condition. The preregistration (including hypotheses and analysis plan), the materials, the data, and the analysis script for this study can be found here: https://osf.io/mbs6r/ (preregistration) and https://osf.io/6jt27/ (for data, analysis script, and description of minor deviations from the preregistration).

### Method

#### Sample

Participants were recruited in the same way as in Study 1. Using the same reasoning for desired sample size while considering that our design consisted of three instead of four conditions, we aimed to have 390 participants. To account for potential missing data, we aimed to collect data from 420 participants.

Our initial sample size consisted of 436 cases. Participants were removed if they (a) had the same IP address (keeping only the first case of each IP address, *n* = 8 cases removed) or (b) had at least three missing values for one of the moral foundation dimensions or did not indicate their ideological identification (*n* = 12 cases removed). Due to the low number of participants excluded due to missing values, a chi-square test of independence could not be conducted. The final sample consisted of 416 participants (55% male, 45% female, *M*_age_ = 34.34, *SD*_age_ = 10.62; liberal target *n* = 134, conservative target *n* = 141, original condition *n* = 141).

#### Design, procedure, and materials

The design was the same as in Study 1, except for two differences. First, we dropped the moderate target condition (i.e., the middle-class condition). Second, we used judgments of 30 MFV as dependent variables instead of the MFQ^5^. We chose the 30 of the 90 MFVs that were most amendable to our manipulation. An example item in the original MFV is, “You see a student stating that her professor is a fool during an afternoon class.” This item was then adapted to “You see a student stating that her [liberal/conservative] professor is a fool during an afternoon class.” This strategy was used for all 30 items (for all items, see https://osf.io/t7h9j/download). The order of these 30 vignettes was randomized. Participants were asked to rate how morally wrong the behavior is on a scale from 1 (*not at all wrong*) to 5 (*extremely wrong*). The six items for each moral foundation were averaged and formed reliable scales in all conditions (care/harm α range [.78, .84]; fairness/cheating α range [.67, .80]; loyalty/betrayal α range [.76, .85]; authority/subversion α range [.82, .85]; sanctity/degradation α range [.85, .88]). Ideological identification was measured and recoded in the same way as Study 1. Our sample leaned liberal (*M* = −0.63, *SD* = 1.72). There was no evidence that the average ideological identification differed in the three conditions (all *p*s > .334).

### Results

#### Confirmatory analysis

**Figure 2.**
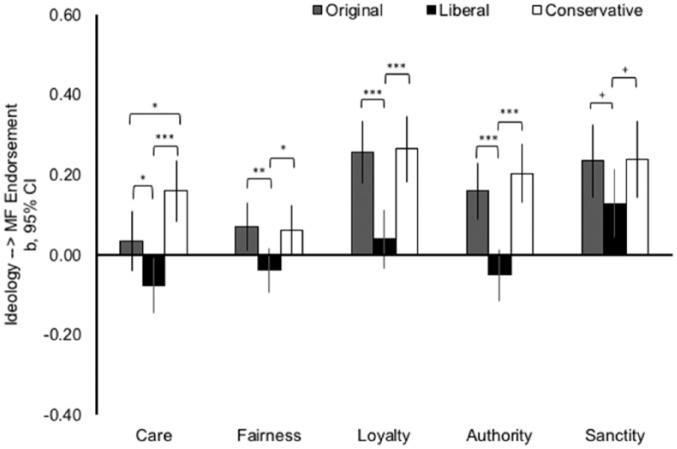
The unstandardized regression coefficients and 95% CIs for the effects of ideological identification in the three experimental conditions on endorsement of the five MF in Study 2. *Note.* Significant differences between the slopes are computed using multiple regression analyses (see main text and supplemental materials). CI = confidence interval; MF = moral foundation. ^†^*p* < .10. *p < .05. **p < .01. ***p < .001.

To test the hypotheses, we followed the same analytic strategy as in Study 1. The results are plotted in Figure 2. Means and standard deviations, as well as full results of the multiple regression analyses underlying Figure 2, are provided in the supplementary materials (Tables SM5 & SM4.1-SM4.5).

##### Individualizing care/harm foundation

We found that all three Ideological Identification × Condition interaction effects were significant. The relationship between ideological identification and endorsement of the individualizing care foundation was significantly more negative in the liberal target condition than in the original condition, interaction *b* = −0.11, *SE* = 0.05, *t*(410) = −2.17, *p* = .030. Furthermore, the relationship between ideological identification and endorsement of the individualizing care foundation was significantly more positive in the conservative target condition than in the original condition, interaction *b* = 0.12, *SE* = 0.05, *t*(410) = 2.27, *p* = .024. Accordingly, the relationship between ideological identification and endorsement of the individualizing care foundation was significantly more negative in the liberal target condition than in the conservative target condition, interaction *b* = −0.24, *SE* = 0.05, *t*(410) = −4.47, *p* < .001. The effect of ideological identification was negative and significant in the liberal target condition, *b* = −0.08, *SE* = 0.04, *t*(410) = −2.21, *p* = .028. The effect was not reliably different from zero in the original condition, *b* = 0.03, *SE* = 0.04, *t*(410) = 0.91, *p* = .363. The effect was positive and significant in the conservative target condition, *b* = 0.16, *SE* = 0.04, *t*(410) = 4.01, *p* < .001.

##### Individualizing fairness/cheating foundation

We found two significant Ideological Identification × Condition interaction effects. The relationship between ideological identification and endorsement of the individualizing fairness foundation was significantly more negative in the liberal target condition than in the two other conditions, interaction *b*s < –0.10, *SE*s = 0.04, *t*(410)s < –2.37, *p*s < .018. The relationship between ideological identification and endorsement of the individualizing fairness foundation was not significantly different in the conservative target condition compared with the original condition, *p* = .856. The effect of ideological identification was negative, but not significant in the liberal target condition, *b* = −0.04, *SE* = 0.03, *t*(410) = −1.39, *p* = .166. Unexpectedly, the effect was positive and significant in the original condition, *b* = 0.07, *SE* = 0.03, *t*(410) = 2.31, *p* = .022. The effect was positive and marginally significant in the conservative target condition, *b* = 0.06, *SE* = 0.03, *t*(410) = 1.95, *p* = .052.

##### Binding loyalty/betrayal foundation

We found two significant Ideological Identification × Condition interaction effects. The relationship between ideological identification and endorsement of the binding loyalty foundation was significantly more negative in the liberal target condition than in the two other conditions, interaction *b*s < –0.21, *SE*s < 0.06, *t*(410)s < –3.97, *p*s < .001. The relationship between ideological identification and endorsement of the binding loyalty foundation was not significantly different in the conservative target condition compared with the original condition, *p =* .877. The effect of ideological identification was strongly positive and significant in the conservative target condition, *b* = 0.26, *SE* = 0.04, *t*(410) = 6.32, *p* < .001, and in the original condition, *b* = 0.26, *SE* = 0.04, *t*(410) = 6.40, *p* < .001. The effect was positive, but not reliably different from zero in the liberal target condition, *b* = 0.04, *SE* = 0.04, *t*(410) = 1.05, *p* = .292.

##### Binding authority/subversion foundation

We found two significant Ideological Identification × Condition interaction effects. The relationship between ideological identification and endorsement of the binding authority foundation was significantly more negative in the liberal target condition than in the two other conditions, interaction *b*s < –0.20, *SE*s = 0.05, *t*(410)s < –4.29, *p*s < .001. The relationship between ideological identification and endorsement of the binding authority foundation was not significantly different in the conservative target condition compared with the original condition, *p =* .389. The effect of ideological identification was strongly positive and significant in the conservative target condition, *b* = 0.20, *SE* = 0.04, *t*(410) = 5.43, *p* < .001, and in the original condition, *b* = 0.16, *SE* = 0.04, *t*(410) = 4.45, *p* < .001. The effect was negative, but not reliably different from zero in the liberal target condition, *b* = −0.05, *SE* = 0.03, *t*(410) = −1.52, *p* = .129.

##### Binding sanctity/degradation foundation

We did not find significant Ideological Identification × Condition interaction effects. However, the relationship between ideological identification and endorsement of the binding sanctity foundation was marginally significantly more negative in the liberal target condition than in the two other conditions, interaction *b*s < –0.10, *SE*s < 0.07, *t*(410)s < –1.65, *p*s < .100. The relationship between ideological identification and endorsement of the binding sanctity foundation was not significantly different in the conservative target condition compared with the original condition, *p =* .944. The effect of ideological identification was strongly positive and significant in the conservative target condition, *b* = 0.24, *SE* = 0.05, *t*(410) = 4.88, *p* < .001, and in the original condition, *b* = 0.23, *SE* = 0.05, *t*(410) = 5.02, *p* < .001. The effect was somewhat smaller in size, but still positive and significant in the liberal target condition, *b* = 0.13, *SE* = 0.04, *t*(410) = 2.97, *p* = .003.

#### Exploratory analyses

For all but the care/harm foundation, we did not replicate the Study 1 finding that participants scored higher in the original condition than in the other two conditions (for full results see supplemental materials). Robustness checks suggest that neither including gender and age as control variables nor testing for nonlinear effects of ideological identification significantly affect our conclusions (for full results see supplemental materials).

### Discussion

The results indicate that the target group affects people’s endorsement of moral foundations. For four of the five foundations, at least two out of the three predicted interaction effects were significant. For the sanctity/degradation foundation, two of the predicted interaction effects were marginally significant. However, not every interaction effect predicted by the political group was found. Here we assess how well each of the hypotheses does when making the predictions that best distinguish the two hypotheses.

1. Effects of ideological identification in the *moderate target* condition (the original condition for the MFV): The political group conflict hypothesis predicts nonsignificant effects of ideological identification across all five foundations, whereas the moral divide hypothesis predicts significant negative effects of ideological identification for the individualizing foundations and significant positive effects of ideological identification for the binding foundations. The data support the moral divide hypothesis for the binding foundations. The effect of ideological identification was nonsignificant for the individualizing care/harm foundation (as predicted by the political group conflict hypothesis) and positive and significant for the individualizing fairness/equality foundation (not predicted by either hypothesis).
2. Effects of ideological identification on endorsement of the binding foundations in the *liberal target* condition: The moral divide hypothesis predicts significant positive effects of ideological identification, whereas the political group conflict hypothesis predicts significant negative effects of ideological identification. Support for the moral divide hypothesis was only found for the binding sanctity foundation, where there was a significant positive association between ideological identification and sanctity endorsement (and no significant interactions). This does not mean that the effects are consistent with the political group conflict hypothesis for the other two binding foundations, as the association between ideological identification and these foundations in the liberal target condition was not significant.
3. Effects of ideological identification on endorsement of the individualizing foundations in the *conservative target* condition: The moral divide hypothesis predicts significant negative effects of ideological identification, whereas the political group conflict hypothesis predicts significant positive effects of ideological identification. The data support the political group conflict hypothesis. The positive association between ideological identification and endorsement of the individualizing foundations in the conservative target group condition was significant for the care/harm foundation and marginally significant for the fairness/cheating foundation.

## General Discussion

Across two studies and two measures of five moral foundations, we found partial support for the political group conflict hypothesis and partial support for the moral divide hypothesis. Differences in moral judgments by liberals and conservatives appear to be caused both by ingroup-versus-outgroup thinking and by genuine differences in moral concerns. The strongest support for the moral divide hypothesis is the finding that conservatives endorsed the binding foundations more strongly than liberals even when a moderate target group was explicitly specified (Study 1). In addition, the lack of significant differences between the moderate target condition and the original condition in Study 1 indicates that the target groups associated with the original MFQ were not clearly perceived as belonging to certain ideological groups. The strongest support for the political group conflict hypothesis is the finding that the effect of ideological identification on the endorsement of the care/harm foundation became positive and significant in the conservative target condition in Study 2.

What do we make out of the lack of strong evidence in support of either theoretical account? As one suggestion, based on the prior literature, we posit that liberals and conservatives have (a) different moral values and (b) group identity interests and care about both of them. When moral foundations and group identity interests conflict, as is the case for liberal authorities and unfairness directed at conservatives, people adjust and update the moral values they express to try and be in line with both. In other words, groups interests and moral values serve as priors that are then adjusted and flexibly updated to try and incorporate new information relevant for the judgment.

Such an integrated account can explain the patterns of data that did not provide strong evidence for either of the two hypotheses. For example, in the conservative target condition in Study 1, we found nonsignificant effects of ideological identification for both the care foundation and the fairness foundation. This is inconsistent with the strong versions of both hypotheses and could occur if both processes were at work but pushing in opposite directions. Another example is that the effect of ideological identification in the political outgroup target condition was typically significantly weakened but mostly did not change directions.

### The Multifaceted Groupishness of Morality

Our results suggest that the political divide is driven by both differences in perceptions of which moral values are considered fundamental for society (e.g., Haidt, 2012) and a political intergroup conflict in which both sides are separated by a strong dislike toward the other group (e.g., Brandt et al., 2014; Mason, 2018). This suggests that morality is groupish, in multiple ways. First, as prior work shows, groups and cultures seem to differ in which values determine moral intuitions and which virtues are admired (Haidt, 2012; Haidt, Koller, & Dias, 1993; Shweder, Much, Mahapatra, & Park, 1997). But beyond that, our studies highlight that people seem to derive their idea of what is right and wrong at least partly from the group memberships of the people involved (see also Cikara, Farnsworth, Harris, & Fiske, 2010; Swann, Gómez, Dovidio, Hart, & Jetten, 2010). Both of these accounts of moral groupishness speak against moral universalism, but in different ways. First, if different groups endorse different moral values to different extents, this contradicts the idea that moral values are universal. However, the political group conflict argument adds to this by showing that people not only differ in their endorsement of different moral values but also in their endorsement of the *same* moral value depending on the groups involved. This is strong evidence for a descriptively relativistic perspective on morality.

There is another possible interpretation of our results. In reaction to scholarly accounts that identify several different moral values (e.g., Haidt, 2007; Shweder et al., 1997), some scholars suggest that morality is not multifaceted, but instead is about a single moral value, namely harm (Gray, Young, & Waytz, 2012). The current findings could be interpreted as support for the special role of another moral value, namely loyalty. That is, the special treatment of political ingroups compared with outgroups. After all, people seem to not only care about harm violations, but they condemn such a harmful act more if it is done to someone who is part of their group. While we do not want to argue that all moral values can be reduced to concerns about loyalty, the relevance of the other values seems to be shaped by thoughts about the status of the target group.

### Methodological Lessons

Furthermore, the current research suggests that the methodological instruments to measure moral values have to be carefully designed to avoid target group confounds. Some have suggested to avoid references to particular political or social groups (Clifford et al., 2015). Others have argued that not referencing a target group might also be problematic as participants could implicitly assume such references (Frimer et al., 2014, Study 2). Therefore, we suggest two additional ways to measure the endorsement of different values. First, scales can reference a broad variety of target groups that are associated with different political ideologies. The representative sample of social groups collected by Koch, Imhoff, Dotsch, Unkelbach, and Alves (2016) might be a good starting point for this approach. In a highly rigorous test of the political group conflict hypothesis, participants would rate the same items for different target groups. However, such a design would require substantial effort from participants (6 items × 5 foundations × ~10 social groups = 300 items) and could create strong consistency pressure that would bias the results. A less comprehensive within-subjects design would use different items for different target groups; however, the perceived ideology and other characteristics of these target groups would need to be carefully balanced. Psychometric research is needed to design ideologically balanced measures of the endorsement of moral values that tap into the same level of the same latent construct while avoiding consistency pressure.

The endorsement of a moral value may most effectively be measured by people’s views on it when the target group is a disliked group. One could argue that it does not require much moral virtue to support fairness or loyalty toward the ingroup, but that such considerations mean much more when it comes to judging moral violations toward the outgroup (Alexander, 2014). Such an approach is already commonly used by researchers studying political intolerance. This “least liked group” method allows participants to select a political group they oppose the most and then complete measures of tolerance for that group (Sullivan, Piereson, & Marcus, 1979). A simplified version of this approach might be to measure the endorsement of moral values only using items that specify the least liked outgroup as the target group.

### Practical Implications

The upside to identifying the addition of a group conflict underpinning to moral foundations is that it can be used to help improve the effectiveness of strategies designed to enable collaboration across the ideological spectrum. For instance, by taking advantage of group identities it may be possible to enhance the moral reframing persuasion process (see Feinberg & Willer, 2015; Voelkel & Feinberg, 2017, for recent work on moral reframing). Prior work finds that conservatives might be more affected by arguments appealing to the binding foundations than liberals. Our findings suggest that the effectiveness of moral reframing might be further increased for liberals and conservatives by connecting a moral argument with the *interest* of their ingroups. Specifying the target group in such ways may not only increase the instrumental value of the argument but also the degree to which the message appeals to the moral values of the reader.

Consistent with this idea, past research indicates that the effect of morally reframed messages on support for certain policies might be mediated by both the fit with readers’ moral values (Feinberg & Willer, 2015) and the extent to which one thinks that the message comes from the ingroup (Wolsko, Ariceaga, & Seiden, 2016). Furthermore, the political group conflict account may also solve one of the ongoing questions in the moral reframing literature: If conservatives rely relatively equally on all five foundations, then why is their support only affected by arguments based on the three binding foundations but not by arguments based on the two individualizing foundations? The political group conflict explanation suggests that the lack of persuasiveness of arguments that appeal to the individualizing foundations might be due to the fact that they often focus on outgroups rather than ingroups. Future research can examine this by testing the effect of moral arguments while manipulating both the moral values underlying the argument and the target groups specified in the argument.

### Limitations and Future Research

There are several noteworthy limitations with the current design. First, our target groups were restricted to a few, ideologically unambiguous groups to maximize the power of the manipulations. An interesting avenue for future research is to extend this research to other target groups (e.g., the most frequently named American groups, cf. Koch et al., 2016). This would not only enable researchers to test the political group conflict hypothesis with a larger sample of groups but also allow testing whether the results for political group conflicts extend to other forms of group conflicts (e.g., between ethnic groups). Past research has provided evidence that nonpolitical group identification can affect participants’ responses to trolley dilemmas (Cikara et al., 2010; Swann et al., 2010). An interesting modification for such studies would be to ask participants not only whether they are part of certain social groups but also to which extent they identify with these groups. This could increase the strength of the political group conflict manipulation. Second, and methodologically, future studies should ensure that their items read naturally for all specified target groups. Special attention should be given to design vignettes that continue to capture the moral relevance and import of the actions that are described. For example, the care/harm foundation was the only foundation for which significant main effects of target condition were found in both conditions. This might be because the liberal and conservative target groups changed the moral relevance of the situation. Third, our samples consisted of American MTurkers. This allowed us to conduct high power tests of our hypotheses with samples that are more diverse than college students (cf. Henry, 2008). However, future research should replicate our results with more representative samples and potentially supporters of other political groups (e.g., libertarians).

The results for the two individualizing foundations in both studies do not consistently indicate that the corresponding moral values are more important for liberals than for conservatives. This is consistent with previous results for the MFV and for the Moral Foundations Sacredness Scale (Clifford et al., 2015, Table 5). It should be noted, however, that the items used in this study referred to emotional care/harm and not to physical care/harm or care/harm toward animals. We think that animals may be an example of a “liberal” target group considering that vegans and vegetarians are associated with progressive views (Koch et al., 2016). Furthermore, in comparison with the results for the binding foundations, the results for the individualizing foundations were less consistent across studies. This requires more research on the influence of difference in the type of measure (Study 1: more abstract moral relevance ratings and more concrete moral judgments vs. Study 2: only more concrete moral judgments) on the relationship between ideological identification and endorsement of individualizing foundations.

### Conclusion

Research on politics in social psychology has been shaped by the idea that liberals and conservatives are divided by the endorsement of different moral foundations (Haidt, 2012). In contrast, recent research suggests that this divide is the result of a political ingroup versus outgroup conflict, which results from similar cognitive processes across the ideological spectrum (Brandt et al., 2014; Frimer et al., 2014). We find evidence that both processes may play a part. On one hand, we provide strong evidence that conservatives endorse the binding foundations more than liberals. On the other hand, we have shown that political group conflicts substantively contribute to the relationship between ideological identification and the endorsement of moral values. These results emphasize that instruments to measure the endorsement of moral values need to include different target groups associated with the whole ideological spectrum and suggest how to enhance the effectiveness of strategies to build a bridge between people from different political camps.

## Supplemental Material

Brandt_OnlineAppendix – Supplemental material for The Effect of Ideological Identification on the Endorsement of Moral Values Depends on the Target Group Click here for additional data file. Supplemental material, Brandt_OnlineAppendix for The Effect of Ideological Identification on the Endorsement of Moral Values Depends on the Target Group by Jan G. Voelkel and Mark J. Brandt in Personality and Social Psychology Bulletin

## Supplemental Material

MFC_-_Supplementary_Materials_-_3rd_Revision_-_Final_Version – Supplemental material for The Effect of Ideological Identification on the Endorsement of Moral Values Depends on the Target Group Click here for additional data file. Supplemental material, MFC_-_Supplementary_Materials_-_3rd_Revision_-_Final_Version for The Effect of Ideological Identification on the Endorsement of Moral Values Depends on the Target Group by Jan G. Voelkel and Mark J. Brandt in Personality and Social Psychology Bulletin

## Notes

1. These target groups were selected based on several empirical studies that measured the perceived ideological identification for various target groups using the same population from which our participants were sampled (American MTurk workers; Brandt, 2017; Koch, Imhoff, Dotsch, Unkelbach, & Alves, 2016). Based on Koch et al.’s ratings, we selected the most liberal group (“liberals”), the most conservative group (“conservatives”), and the most moderate group (“middle class”). The scores of liberals and conservatives were similarly close to the extremes of the scales indicating that liberals and conservatives are perceived as being similarly ideologically extreme (identical conclusions are made from Brandt’s [2017] data).

2. The correlation of .34 is the correlation between ideology and support for the authority foundation in Graham, Haidt, and Nosek (2009).

3. Two items from the moral judgments part of the original Moral Foundation Questionnaire (MFQ) were replaced with two other moral judgments items from a previous version of the MFQ (Graham et al., 2009) because these items did not allow for the appropriate inclusion of the target groups. First, “Men and women each have different roles to play in society” was replaced by “When the government makes laws, those laws should always respect the traditions and heritage of the country.” Second, “Chastity is an important and valuable virtue” was replaced by “The government should try to help people live virtuously and avoid sin.”

4. For all items, see https://osf.io/w498u/download.

5. For all items, see https://osf.io/t7h9j/download.
