# Supplementary material for: The Effect of Ideological Identification on the Endorsement of Moral Values Depends on the Target Group
Source: Pers Soc Psychol Bull. 2018 Oct 13;45(6):851–63. doi: 10.1177/0146167218798822 (PMC6526610; doi:10.1177/0146167218798822)
Supplement: Brandt_OnlineAppendix – Supplemental material for The Effect of Ideological Identification on the Endorsement of Moral Values Depends on the Target Group [file Brandt_OnlineAppendix.pdf]

MFC - Study 1

Survey Flow

Standard: Consent Form (1 Question)

BlockRandomizer: 1 - Evenly Present Elements

EmbeddedData
Condition = Original
EmbeddedData
Condition = Liberal
EmbeddedData
Condition = Conservative
EmbeddedData
Condition = Neutral

Branch: New Branch
If
If Condition Is Equal to Original

EmbeddedData
Group_C1 = someone
Group_C2 = someone weak or vulnerable
Group_C3 = cruel
Group_C4 = those who are suffering is the most crucial virtue
Group_C5 = a defenseless animal
Group_C6 = a human being
Group_F1 = some people
Group_F2 = unfairly
Group_F3 = someone was denied his or her rights
Group_F4 = the number one principle should be ensuring that everyone is treated fairly
Group_F5 = is the most important requirement for a society
Group_F6 = that rich children inherit a lot of money while poor children inherit nothing
Group_L1 = his or her country
Group_L2 = his or her group
Group_L3 = loyalty
Group_L4 = my country's history
Group_L5 = their family members
Group_L6 = It is more important to be a team player than to express oneself
Group_A1 = authority
Group_A2 = society
Group_A3 = disorder
Group_A4 = authority
Group_A5 = the country
Group_A6 = were a soldier and disagreed with my commanding officer’s orders
Group_S1 = decency

Group_S2 = disgusting
Group_S3 = God would approve of
Group_S4 = are
Group_S5 = that
Group_S6 = virtuously and avoid sin

Branch: New Branch
If
If Condition Is Equal to Liberal

EmbeddedData
Group_C1 = a liberal person
Group_C2 = a weak or vulnerable liberal person
Group_C3 = cruel to a liberal person
Group_C4 = liberal people who are suffering is a crucial virtue
Group_C5 = a liberal person
Group_C6 = a liberal person
Group_F1 = people who are liberal
Group_F2 = unfairly towards a liberal person
Group_F3 = a liberal person was denied his or her rights
Group_F4 = an important principle should be ensuring that people who are liberal are treated fairly
Group_F5 = for people who are liberal is an important requirement for a society
Group_F6 = when people who are liberal have fewer resources than other groups
Group_L1 = liberals
Group_L2 = liberals
Group_L3 = loyalty to liberals
Group_L4 = the history of liberals
Group_L5 = people who are liberal
Group_L6 = When in a group of liberals, a person should rather be a team player than express themselves
Group_A1 = liberal authorities
Group_A2 = people who are liberal
Group_A3 = disorder that disrupted people who are liberal
Group_A4 = liberal authorities
Group_A5 = people who are liberal
Group_A6 = disagreed with the orders of liberal authorities
Group_S1 = decency of people who are liberal
Group_S2 = people who are liberal would find disgusting
Group_S3 = people who are liberal would find spiritually pure
Group_S4 = people who are liberal find
Group_S5 = that people who are liberal think that
Group_S6 = in a way that people who are liberal would consider as virtuous

Branch: New Branch

If

If Condition Is Equal to Conservative

EmbeddedData

Group_C1 = a conservative person
Group_C2 = a weak or vulnerable conservative person
Group_C3 = cruel to a conservative person
Group_C4 = conservative people who are suffering is a crucial virtue
Group_C5 = a conservative person
Group_C6 = a conservative person
Group_F1 = people who are conservative
Group_F2 = unfairly towards a conservative person
Group_F3 = a conservative person was denied his or her rights
Group_F4 = an important principle should be ensuring that people who are conservative are treated fairly
Group_F5 = for people who are conservative is an important requirement for a society
Group_F6 = when people who are conservative have fewer resources than other groups
Group_L1 = conservatives
Group_L2 = conservatives
Group_L3 = loyalty to conservatives
Group_L4 = the history of conservatives
Group_L5 = people who are conservative
Group_L6 = When in a group of conservatives, a person should rather be a team player than express themselves
Group_A1 = conservative authorities
Group_A2 = people who are conservative
Group_A3 = disorder that disrupted people who are conservative
Group_A4 = conservative authorities
Group_A5 = people who are conservative
Group_A6 = disagreed with the orders of conservative authorities
Group_S1 = decency of people who are conservative
Group_S2 = people who are conservative would find disgusting
Group_S3 = people who are conservative would find spiritually pure
Group_S4 = people who are conservative find
Group_S5 = that people who are conservative think that
Group_S6 = in a way that people who are conservative would consider as virtuous

Branch: New Branch

If

If Condition Is Equal to Neutral

EmbeddedData

Group_C1 = a person from the middle class
Group_C2 = a weak or vulnerable person from the middle class
Group_C3 = cruel to a person from the middle class
Group_C4 = people from the middle class who are suffering is a crucial virtue
Group_C5 = a person from the middle class
Group_C6 = a person from the middle class
Group_F1 = people who are from the middle class
Group_F2 = unfairly towards a person from the middle class
Group_F3 = a person from the middle class was denied his or her rights
Group_F4 = an important principle should be ensuring that people who are from the middle class are treated fairly
Group_F5 = for people who are from the middle class is an important requirement for a society
Group_F6 = when people who are from the middle class have fewer resources than other groups
Group_L1 = middle class people
Group_L2 = middle class people
Group_L3 = loyalty to middle class people
Group_L4 = the history of middle class people
Group_L5 = people who are from the middle class
Group_L6 = When in a group of middle class people, a person should rather be a team player than express themselves
Group_A1 = authorities who are middle class people
Group_A2 = people who are from the middle class
Group_A3 = disorder that disrupted people who are from the middle class
Group_A4 = authorities who are middle class people
Group_A5 = people who are from the middle class
Group_A6 = disagreed with the orders of authorities who are middle class people
Group_S1 = decency of people who are from the middle class
Group_S2 = people who are from the middle class would find disgusting
Group_S3 = people who are from the middle class would find spiritually pure
Group_S4 = people who are from the middle class find
Group_S5 = that people who are from the middle class think that
Group_S6 = in a way that people who are from the middle class would consider as virtuous

Standard: Introduction (1 Question)
Standard: MFQ (32 Questions)
Block: Demograpics (5 Questions)
Standard: Debriefing (3 Questions)

EndSurvey:

Page Break

Start of Block: Consent Form

CF **Consent to Participate in Research**

Description
If you choose to participate, you will be asked to indicate your opinions about some statements.
Benefits
There is no direct benefit to you from this research. We hope that this research will benefit others and society by increasing scientific knowledge. You will be paid $0.50 for participating in this study.

Risks/Discomforts
There is a small risk that you may become uncomfortable during the study. If at any point during the study proceedings you become uncomfortable, you may elect to withdraw your consent to participate without consequences to you. To withdraw your consent, simply close this browser window. If any feelings of discomfort emerge you are free to contact the lead investigator with any questions or concerns using the email address listed at the bottom of this consent form.
Confidentiality
All of the information that we obtain from your session is anonymous. There will be no record that links the data collected from you with any personal data from which you could be identified (e.g., your name, address, email etc.). These anonymized data may be made available to researchers via accessible data repositories and possibly used for novel purposes. The anonymized data will be stored for at least 10 years.

Voluntary Nature of Participation
Your decision to participate in this study is completely voluntary. Thus, you may refuse to join the study or terminate your participation at any time without negative consequences. Should you wish to terminate your participation at any point, you only need to close the browser window.

Contact Information
If you have any questions or concerns about this study, you may contact the contact persons for this study: [REDACTED] or [REDACTED].
Consent
If you consent to participate in this study, please click "I agree to participate". If you do not consent to participate, please close the browser window.

- I agree to participate. (1)

End of Block: Consent Form

Start of Block: Introduction

intro We are interested in the types of things people think are relevant for making a moral decision.

On the next two pages, you will answer 30 questions about the types of things you consider relevant when judging if something is moral or immoral. These include the type of actions, behaviors, and people involved.

You may find some more or less relevant than others. There is no right answer and all of your responses are anonymous, so please give us your honest answer.

End of Block: Introduction

Start of Block: MFQ

MFQ_Intro1 When you decide whether something is right or wrong, to what extent are the following considerations relevant to your thinking?

X→ X→

MFQ_C1 Whether or not ${e://Field/Group_C1} suffered emotionally

- O Not at all relevant 0 (0)
- O Not very relevant 1 (1)
- O Slightly relevant 2 (2)
- O Somewhat relevant 3 (3)
- O Very relevant 4 (4)
- O Extremely relevant 5 (5)

X→ X→

MFQ_F1 Whether or not ${e://Field/Group_F1} were treated differently from others

- Not at all relevant 0 (0)
- Not very relevant 1 (1)
- Slightly relevant 2 (2)
- Somewhat relevant 3 (3)
- Very relevant 4 (4)
- Extremely relevant 5 (5)

MFQ_L1 Whether or not someone's action showed love for ${e://Field/Group_L1}

- Not at all relevant 0 (0)
- Not very relevant 1 (1)
- Slightly relevant 2 (2)
- Somewhat relevant 3 (3)
- Very relevant 4 (4)
- Extremely relevant 5 (5)

MFQ_A1 Whether   or not someone showed a lack of respect for ${e://Field/Group_A1}

- O Not at all relevant 0 (0)
- O Not very relevant 1 (1)
- O Slightly relevant 2 (2)
- O Somewhat relevant 3 (3)
- O Very relevant 4 (4)
- O Extremely relevant 5 (5)

X→ X→

MFQ_S1 Whether   or not someone violated standards of purity and ${e://Field/Group_S1}

- O Not at all relevant 0 (0)
- O Not very relevant 1 (1)
- O Slightly relevant 2 (2)
- O Somewhat relevant 3 (3)
- O Very relevant 4 (4)
- O Extremely relevant 5 (5)

X→ X→

MFQ_C2 Whether   or not someone cared for ${e://Field/Group_C2}

- Not at all relevant 0 (0)
- Not very relevant 1 (1)
- Slightly relevant 2 (2)
- Somewhat relevant 3 (3)
- Very relevant 4 (4)
- Extremely relevant 5 (5)

MFQ_F2 Whether   or not someone acted ${e://Field/Group_F2}

- Not at all relevant 0 (0)
- Not very relevant 1 (1)
- Slightly relevant 2 (2)
- Somewhat relevant 3 (3)
- Very relevant 4 (4)
- Extremely relevant 5 (5)

MFQ_L2 Whether or not someone did something to betray ${e://Field/Group_L2}

- Not at all relevant 0 (0)
- Not very relevant 1 (1)
- Slightly relevant 2 (2)
- Somewhat relevant 3 (3)
- Very relevant 4 (4)
- Extremely relevant 5 (5)

X→ X→

MFQ_A2 Whether or not someone conformed to the traditions of ${e://Field/Group_A2}

- Not at all relevant 0 (0)
- Not very relevant 1 (1)
- Slightly relevant 2 (2)
- Somewhat relevant 3 (3)
- Very relevant 4 (4)
- Extremely relevant 5 (5)

X→ X→

MFQ_S2 Whether   or not someone did something ${e://Field/Group_S2}

- Not at all relevant 0 (0)
- Not very relevant 1 (1)
- Slightly relevant 2 (2)
- Somewhat relevant 3 (3)
- Very relevant 4 (4)
- Extremely relevant 5 (5)

MFQ_C3 Whether   or not someone was ${e://Field/Group_C3}

- Not at all relevant 0 (0)
- Not very relevant 1 (1)
- Slightly relevant 2 (2)
- Somewhat relevant 3 (3)
- Very relevant 4 (4)
- Extremely relevant 5 (5)

MFQ_F3 Whether or not ${e://Field/Group_F3}

- Not at all relevant 0 (0)
- Not very relevant 1 (1)
- Slightly relevant 2 (2)
- Somewhat relevant 3 (3)
- Very relevant 4 (4)
- Extremely relevant 5 (5)

MFQ_L3 Whether or not someone showed a lack of ${e://Field/Group_L3}

- Not at all relevant 0 (0)
- Not very relevant 1 (1)
- Slightly relevant 2 (2)
- Somewhat relevant 3 (3)
- Very relevant 4 (4)
- Extremely relevant 5 (5)

MFQ_A3 Whether or not an action caused chaos or ${e://Field/Group_A3}

- Not at all relevant 0 (0)
- Not very relevant 1 (1)
- Slightly relevant 2 (2)
- Somewhat relevant 3 (3)
- Very relevant 4 (4)
- Extremely relevant 5 (5)

X→ X→

MFQ_S3 Whether or not someone acted in a way that ${e://Field/Group_S3}

- Not at all relevant 0 (0)
- Not very relevant 1 (1)
- Slightly relevant 2 (2)
- Somewhat relevant 3 (3)
- Very relevant 4 (4)
- Extremely relevant 5 (5)

Page Break

MFQ_Intro2 Please read the following sentences and indicate your agreement or disagreement.

MFQ_C4 Compassion for ${e://Field/Group_C4}.

- Strongly disagree 0 (0)
- Moderately disagree 1 (1)
- Slightly disagree 2 (2)
- Slightly agree 3 (3)
- Moderately agree 4 (4)
- Strongly agree 5 (5)

MFQ_F4 When the government makes laws, ${e://Field/Group_F4}.

- Strongly disagree 0 (0)
- Moderately disagree 1 (1)
- Slightly disagree 2 (2)
- Slightly agree 3 (3)
- Moderately agree 4 (4)
- Strongly agree 5 (5)

MFQ_L4 I am proud of ${e://Field/Group_L4}.

- O Strongly disagree 0 (0)
- O Moderately disagree 1 (1)
- O Slightly disagree 2 (2)
- O Slightly agree 3 (3)
- O Moderately agree 4 (4)
- O Strongly agree 5 (5)

X→ X→

MFQ_A4 Respect for ${e://Field/Group_A4} is something all children need to learn.

- O Strongly disagree 0 (0)
- O Moderately disagree 1 (1)
- O Slightly disagree 2 (2)
- O Slightly agree 3 (3)
- O Moderately agree 4 (4)
- O Strongly agree 5 (5)

X→ X→

MFQ_S4 People should not do things that ${e://Field/Group_S4} disgusting, even if no one is harmed.

- Strongly disagree 0 (0)
- Moderately disagree 1 (1)
- Slightly disagree 2 (2)
- Slightly agree 3 (3)
- Moderately agree 4 (4)
- Strongly agree 5 (5)

MFQ_C5 One of the worst things a person could do is hurt ${e://Field/Group_C5}.

- Strongly disagree 0 (0)
- Moderately disagree 1 (1)
- Slightly disagree 2 (2)
- Slightly agree 3 (3)
- Moderately agree 4 (4)
- Strongly agree 5 (5)

MFQ_F5 Justice ${e://Field/Group_F5}.

- Strongly disagree 0 (0)
- Moderately disagree 1 (1)
- Slightly disagree 2 (2)
- Slightly agree 3 (3)
- Moderately agree 4 (4)
- Strongly agree 5 (5)

X→ X→

MFQ_L5 People should be loyal to ${e://Field/Group_L5}, even when they have done something wrong.

- Strongly disagree 0 (0)
- Moderately disagree 1 (1)
- Slightly disagree 2 (2)
- Slightly agree 3 (3)
- Moderately agree 4 (4)
- Strongly agree 5 (5)

X→ X→

MFQ_A5 When   the government makes laws, those laws should always respect the traditions and heritage of ${e://Field/Group_A5}.

- Strongly disagree 0 (0)
- Moderately disagree 1 (1)
- Slightly disagree 2 (2)
- Slightly agree 3 (3)
- Moderately agree 4 (4)
- Strongly agree 5 (5)

MFQ_S5 I would   call some acts wrong on the grounds ${e://Field/Group_S5} they are unnatural.

- Strongly disagree 0 (0)
- Moderately disagree 1 (1)
- Slightly disagree 2 (2)
- Slightly agree 3 (3)
- Moderately agree 4 (4)
- Strongly agree 5 (5)

MFQ_C6 It can never be right to kill ${e://Field/Group_C6}.

- O Strongly disagree 0 (0)
- O Moderately disagree 1 (1)
- O Slightly disagree 2 (2)
- O Slightly agree 3 (3)
- O Moderately agree 4 (4)
- O Strongly agree 5 (5)

MFQ_F6 I think it's morally wrong ${e://Field/Group_F6}.

- O Strongly disagree 0 (0)
- O Moderately disagree 1 (1)
- O Slightly disagree 2 (2)
- O Slightly agree 3 (3)
- O Moderately agree 4 (4)
- O Strongly agree 5 (5)

MFQ_L6 ${e://Field/Group_L6}.

- O Strongly disagree 0 (0)
- O Moderately disagree 1 (1)
- O Slightly disagree 2 (2)
- O Slightly agree 3 (3)
- O Moderately agree 4 (4)
- O Strongly agree 5 (5)

X→ X→

MFQ_A6 If I ${e://Field/Group_A6}, I would obey anyway because that is my duty.

- O Strongly disagree 0 (0)
- O Moderately disagree 1 (1)
- O Slightly disagree 2 (2)
- O Slightly agree 3 (3)
- O Moderately agree 4 (4)
- O Strongly agree 5 (5)

X→ X→

MFQ_S6 The   government should try to help people live ${e://Field/Group_S6}.

- O Strongly disagree 0 (0)
- O Moderately disagree 1 (1)
- O Slightly disagree 2 (2)
- O Slightly agree 3 (3)
- O Moderately agree 4 (4)
- O Strongly agree 5 (5)

End of Block: MFQ

Start of Block: Demograpics

Intro_Demo Please answer the following questions.

Gender Please indicate your gender:

- O Female (1)
- O Male (2)
- O Other: (3) ____

Age Please indicate your age:

____

Ethnicity Which ethnic group do you identify most with? Choose all that apply.

- ▢ Caucasian/European/European American (1)
- ▢ Asian/Asian American (2)
- ▢ Indian/Indian American (3)
- ▢ African/African American (4)
- ▢ Hispanic/Latino (5)
- ▢ Native American (6)
- ▢ Prefer not to answer (7)
- ▢ Other (8) ____

Ideology Generally speaking, do you usually think of yourself as conservative, moderate, or liberal?

- ○ Very conservative (1)
- ○ Conservative (2)
- ○ Moderate, lean conservative (3)
- ○ Moderate (4)
- ○ Moderate, lean liberal (5)
- ○ Liberal (6)
- ○ Very liberal (7)

End of Block: Demograpics

Start of Block: Debriefing

Debriefing Thank you for participating in this study on moral values and social groups!

In this study, we were interested in whether people with different political beliefs endorse certain moral values more depending on what kind of group the moral value refers to. Some people think that liberals and conservatives have very different values, but we think that this might be because some measures of moral values make reference to specific groups. Our study is looking at this question.

Your participation will help us increase our understanding of the similarities and dissimilarities between liberals and conservatives with regard to their moral value systems and in the future help us develop methods to help people with different worldviews get along.

Please do not share the purpose of the study with other people who might participate in this study. This could make the results of the study less accurate and useful.

Thanks!

Page Break

Code The study is now finished. Thank you for your participation.

To get paid, please go to the next page where you will see your confirmation code. Use this code on the MTurk page where you found the link to the study.

Comments Do you have any comments for us? Please let us know

____

____

____

____

____

End of Block: Debriefing

MFC - Study 2

Survey Flow

Standard: Consent Form (1 Question)

BlockRandomizer: 1 - Evenly Present Elements

EmbeddedData
Condition = Original
EmbeddedData
Condition = Liberal
EmbeddedData
Condition = Conservative

Branch: New Branch
If
If Condition Is Equal to Original

EmbeddedData
Group_C1 = an amputee
Group_C2 = woman
Group_C3 = the janitor
Group_C4 = brother
Group_C5 = an obese woman
Group_C6 = disabled co-worker
Group_F1 = tenant
Group_F2 = strangers
Group_F3 = judge
Group_F4 = employee
Group_F5 = student
Group_F6 = politician
Group_L1a = mayor
Group_L1b = town
Group_L2a = the
Group_L2b = Americans
Group_L3a = man
Group_L3b = family
Group_L4a = Hollywood
Group_L4b = US
Group_L5a = American
Group_L5b = world
Group_L6a = Secretary
Group_L6b = US
Group_A1 = father's
Group_A2 = teacher
Group_A3 = boss'
Group_A4 = military service
Group_A5 = mayor's

Group_A6 = professor
Group_S1 = man
Group_S2 = man
Group_S3a = homosexual
Group_S3b = gay bar
Group_S4 = man
Group_S5 = cousins
Group_S6 = man

Branch: New Branch
If
If Condition Is Equal to Liberal

EmbeddedData
Group_C1 = a person who is liberal
Group_C2 = woman is liberal
Group_C3 = a liberal
Group_C4 = liberal brother
Group_C5 = a woman who is liberal
Group_C6 = co-worker who is liberal
Group_F1 = tenant who is liberal
Group_F2 = strangers who are liberal
Group_F3 = conservative judge
Group_F4 = employee who is conservative
Group_F5 = liberal student
Group_F6 = conservative politician
Group_L1a = liberal mayor
Group_L1b = town which is conservative
Group_L2a = a liberal
Group_L2b = liberal Americans
Group_L3a = man who is liberal
Group_L3b = liberal family
Group_L4a = liberal Hollywood
Group_L4b = US during the term of a liberal president
Group_L5a = American who is liberal
Group_L5b = world during the term of a liberal president
Group_L6a = liberal Secretary
Group_L6b = US during the term of a liberal president
Group_A1 = liberal father's
Group_A2 = teacher who is liberal
Group_A3 = liberal boss'
Group_A4 = support for liberal policies
Group_A5 = liberal mayor's
Group_A6 = liberal professor
Group_S1 = man who is liberal

Group_S2 = man who is liberal
Group_S3a = liberal
Group_S3b = bar
Group_S4 = man who is liberal
Group_S5 = cousins who are liberal
Group_S6 = man who is liberal

Branch: New Branch
If
If Condition Is Equal to Conservative

EmbeddedData
Group_C1 = a person who is conservative
Group_C2 = woman is conservative
Group_C3 = a conservative
Group_C4 = conservative brother
Group_C5 = a woman who is conservative
Group_C6 = co-worker who is conservative
Group_F1 = tenant who is conservative
Group_F2 = strangers who are conservative
Group_F3 = liberal judge
Group_F4 = employee who is liberal
Group_F5 = conservative student
Group_F6 = liberal politician
Group_L1a = conservative mayor
Group_L1b = town which is liberal
Group_L2a = a conservative
Group_L2b = conservative Americans
Group_L3a = man who is conservative
Group_L3b = conservative family
Group_L4a = conservative Hollywood
Group_L4b = US during the term of a conservative president
Group_L5a = American who is conservative
Group_L5b = world during the term of a conservative president
Group_L6a = conservative Secretary
Group_L6b = US during the term of a conservative president
Group_A1 = conservative father's
Group_A2 = teacher who is conservative
Group_A3 = conservative boss'
Group_A4 = support for conservative policies
Group_A5 = conservative mayor's
Group_A6 = conservative professor
Group_S1 = man who is conservative
Group_S2 = man who is conservative
Group_S3a = conservative

Group_S3b = bar
Group_S4 = man who is conservative
Group_S5 = cousins who are conservative
Group_S6 = man who is conservative

Standard: Introduction (1 Question)
Standard: Moral Vignettes (31 Questions)
Block: Demograpics (5 Questions)
Standard: Debriefing (3 Questions)

EndSurvey:

Page Break

Start of Block: Consent Form

CF **Consent to Participate in Research**

Description
If you choose to participate, you will be asked to indicate your opinions about some statements.
Benefits
There is no direct benefit to you from this research. We hope that this research will benefit others and society by increasing scientific knowledge. You will be paid $0.50 for participating in this study.

Risks/Discomforts
There is a small risk that you may become uncomfortable during the study. If at any point during the study proceedings you become uncomfortable, you may elect to withdraw your consent to participate without consequences to you. To withdraw your consent, simply close this browser window. If any feelings of discomfort emerge you are free to contact the lead investigator with any questions or concerns using the email address listed at the bottom of this consent form.
Confidentiality
All of the information that we obtain from your session is anonymous. There will be no record that links the data collected from you with any personal data from which you could be identified (e.g., your name, address, email etc.). These anonymized data may be made available to researchers via accessible data repositories and possibly used for novel purposes. The anonymized data will be stored for at least 10 years.

Voluntary Nature of Participation
Your decision to participate in this study is completely voluntary. Thus, you may refuse to join the study or terminate your participation at any time without negative consequences. Should you wish to terminate your participation at any point, you only need to close the browser window.

Contact Information
If you have any questions or concerns about this study, you may contact the contact persons for this study: [REDACTED] or [REDACTED].
Consent
If you consent to participate in this study, please click "I agree to participate". If you do not consent to participate, please close the browser window.

- O I agree to participate. (1)

End of Block: Consent Form

Start of Block: Introduction

Intro1 We are interested in how people judge different behaviors in terms of their moral wrongness.

On the next page, you will read about 30 behaviors and rate how morally wrong these behaviors are. The description of the behaviors include certain actions and people involved.

You may find some more or less morally wrong than others. All of your responses are anonymous, so please give us your honest answer.

End of Block: Introduction

Start of Block: Moral Vignettes

Intro2 How morally wrong are the following behaviors?

MV_C1 You see a teenage boy chuckling at ${e://Field/Group_C1} he passes by while on the subway.

- Not at all wrong 1 (1)
- Not too wrong 2 (2)
- Somewhat wrong 3 (3)
- Very wrong 4 (4)
- Extremely wrong 5 (5)

MV_C2 You see a man   quickly canceling a blind date as soon as he sees the ${e://Field/Group_C2}.

- Not at all wrong 1 (1)
- Not too wrong 2 (2)
- Somewhat wrong 3 (3)
- Very wrong 4 (4)
- Extremely wrong 5 (5)

MV_C3 You see a girl   laughing when she realizes her friend's dad is ${e://Field/Group_C3}.

- Not at all wrong 1 (1)
- Not too wrong 2 (2)
- Somewhat wrong 3 (3)
- Very wrong 4 (4)
- Extremely wrong 5 (5)

MV_C4 You see a boy making fun of his ${e://Field/Group_C4} for getting dumped by his girlfriend.

- Not at all wrong 1 (1)
- Not too wrong 2 (2)
- Somewhat wrong 3 (3)
- Very wrong 4 (4)
- Extremely wrong 5 (5)

MV_C5 You see a woman clearly avoiding sitting next to ${e://Field/Group_C5} on the bus.

- Not at all wrong 1 (1)
- Not too wrong 2 (2)
- Somewhat wrong 3 (3)
- Very wrong 4 (4)
- Extremely wrong 5 (5)

MV_C6 You see a man laughing at a ${e://Field/Group_C6} while at an office softball game.

O Not at all wrong 1 (1)

O Not too wrong 2 (2)

O Somewhat wrong 3 (3)

O Very wrong 4 (4)

O Extremely wrong 5 (5)

MV_F1 You see a ${e://Field/Group_F1} bribing a landlord to be the first to get their apartment repainted.

O Not at all wrong 1 (1)

O Not too wrong 2 (2)

O Somewhat wrong 3 (3)

O Very wrong 4 (4)

O Extremely wrong 5 (5)

MV_F2 You see someone cheating in a card game while playing with a group of ${e://Field/Group_F2}.

- Not at all wrong 1 (1)
- Not too wrong 2 (2)
- Somewhat wrong 3 (3)
- Very wrong 4 (4)
- Extremely wrong 5 (5)

X→ X→

MV_F3 You see a ${e://Field/Group_F3} taking on a criminal case although he is friends with the defendant.

- Not at all wrong 1 (1)
- Not too wrong 2 (2)
- Somewhat wrong 3 (3)
- Very wrong 4 (4)
- Extremely wrong 5 (5)

X→ X→

MV_F4 You see an ${e://Field/Group_F4} lying about how many hours she worked during the week.

- Not at all wrong 1 (1)
- Not too wrong 2 (2)
- Somewhat wrong 3 (3)
- Very wrong 4 (4)
- Extremely wrong 5 (5)

MV_F5 You see a professor giving a bad grade to a ${e://Field/Group_F5} just because he dislikes him.

- Not at all wrong 1 (1)
- Not too wrong 2 (2)
- Somewhat wrong 3 (3)
- Very wrong 4 (4)
- Extremely wrong 5 (5)

MV_F6 You see a ${e://Field/Group_F6} using federal tax dollars to build an extension on his home.

- Not at all wrong 1 (1)
- Not too wrong 2 (2)
- Somewhat wrong 3 (3)
- Very wrong 4 (4)
- Extremely wrong 5 (5)

MV_L1 You see a ${e://Field/Group_L1a} saying that the neighboring ${e://Field/Group_L1b} is a much better town.

- Not at all wrong 1 (1)
- Not too wrong 2 (2)
- Somewhat wrong 3 (3)
- Very wrong 4 (4)
- Extremely wrong 5 (5)

MV_L2 You see ${e://Field/Group_L2a} US Ambassador joking in Great Britain about the stupidity of ${e://Field/Group_L2b}.

- Not at all wrong 1 (1)
- Not too wrong 2 (2)
- Somewhat wrong 3 (3)
- Very wrong 4 (4)
- Extremely wrong 5 (5)

MV_L3 You see a ${e://Field/Group_L3a} leaving his ${e://Field/Group_L3b} business to go work for their main competitor.

- Not at all wrong 1 (1)
- Not too wrong 2 (2)
- Somewhat wrong 3 (3)
- Very wrong 4 (4)
- Extremely wrong 5 (5)

MV_L4 You see a ${e://Field/Group_L4a} star agreeing with a foreign dictator's denunciation of the ${e://Field/Group_L4b}.

- Not at all wrong 1 (1)
- Not too wrong 2 (2)
- Somewhat wrong 3 (3)
- Very wrong 4 (4)
- Extremely wrong 5 (5)

MV_L5 You see an ${e://Field/Group_L5a} telling foreigners that the US is an evil force in the ${e://Field/Group_L5b}.

- Not at all wrong 1 (1)
- Not too wrong 2 (2)
- Somewhat wrong 3 (3)
- Very wrong 4 (4)
- Extremely wrong 5 (5)

MV_L6 You see a former ${e://Field/Group_L6a} of State publicly giving up his citizenship to the ${e://Field/Group_L6b}.

- Not at all wrong 1 (1)
- Not too wrong 2 (2)
- Somewhat wrong 3 (3)
- Very wrong 4 (4)
- Extremely wrong 5 (5)

MV_A1 You see a girl ignoring her ${e://Field/Group_A1} orders by taking the car after her curfew.

- Not at all wrong 1 (1)
- Not too wrong 2 (2)
- Somewhat wrong 3 (3)
- Very wrong 4 (4)
- Extremely wrong 5 (5)

MV_A2 You see a girl   repeatedly interrupting her ${e://Field/Group_A2} as he explains a new concept.

- Not at all wrong 1 (1)
- Not too wrong 2 (2)
- Somewhat wrong 3 (3)
- Very wrong 4 (4)
- Extremely wrong 5 (5)

MV_A3 You see an employee   trying to undermine all of her ${e://Field/Group_A3} ideas in front of others.

- Not at all wrong 1 (1)
- Not too wrong 2 (2)
- Somewhat wrong 3 (3)
- Very wrong 4 (4)
- Extremely wrong 5 (5)

MV_A4 You see a boy turning up the TV as his father talks about his ${e://Field/Group_A4}.

- Not at all wrong 1 (1)
- Not too wrong 2 (2)
- Somewhat wrong 3 (3)
- Very wrong 4 (4)
- Extremely wrong 5 (5)

MV_A5 You see a staff member talking loudly and interrupting the ${e://Field/Group_A5} speech to the public.

- Not at all wrong 1 (1)
- Not too wrong 2 (2)
- Somewhat wrong 3 (3)
- Very wrong 4 (4)
- Extremely wrong 5 (5)

MV_A6 You see a student stating that her ${e://Field/Group_A6} is a fool during an afternoon class.

- Not at all wrong 1 (1)
- Not too wrong 2 (2)
- Somewhat wrong 3 (3)
- Very wrong 4 (4)
- Extremely wrong 5 (5)

MV_S1 You see a ${e://Field/Group_S1} having sex with a frozen chicken before cooking it for dinner.

- Not at all wrong 1 (1)
- Not too wrong 2 (2)
- Somewhat wrong 3 (3)
- Very wrong 4 (4)
- Extremely wrong 5 (5)

MV_S2 You see a drunk elderly ${e://Field/Group_S2} offering to have oral sex with anyone in the bar.

- Not at all wrong 1 (1)
- Not too wrong 2 (2)
- Somewhat wrong 3 (3)
- Very wrong 4 (4)
- Extremely wrong 5 (5)

MV_S3 You see a ${e://Field/Group_S3a} in a ${e://Field/Group_S3b} offering sex to anyone who buys him a drink.

- Not at all wrong 1 (1)
- Not too wrong 2 (2)
- Somewhat wrong 3 (3)
- Very wrong 4 (4)
- Extremely wrong 5 (5)

MV_S4 You see a ${e://Field/Group_S4} searching through the trash to find women's discarded underwear.

- Not at all wrong 1 (1)
- Not too wrong 2 (2)
- Somewhat wrong 3 (3)
- Very wrong 4 (4)
- Extremely wrong 5 (5)

MV_S5 You see two first ${e://Field/Group_S5} getting married to each other in an elaborate wedding.

- Not at all wrong 1 (1)
- Not too wrong 2 (2)
- Somewhat wrong 3 (3)
- Very wrong 4 (4)
- Extremely wrong 5 (5)

MV_S6 You see a single ${e://Field/Group_S6} ordering an inflatable sex doll that looks like his secretary.

- Not at all wrong 1 (1)
- Not too wrong 2 (2)
- Somewhat wrong 3 (3)
- Very wrong 4 (4)
- Extremely wrong 5 (5)

End of Block: Moral Vignettes

Start of Block: Demograpics

Intro3 Please answer the following questions.

Gender Please indicate your gender:

- Female (1)
- Male (2)
- Other: (3) ____

Age Please indicate your age:

____

Ethnicity Which ethnic group do you identify most with? Choose all that apply.

- ▢ Caucasian/European/European American (1)
- ▢ Asian/Asian American (2)
- ▢ Indian/Indian American (3)
- ▢ African/African American (4)
- ▢ Hispanic/Latino (5)
- ▢ Native American (6)
- ▢ Prefer not to answer (7)
- ▢ Other (8) ____

Ideology Generally speaking, do you usually think of yourself as conservative, moderate, or liberal?

- O Very conservative (1)
- O Conservative (2)
- O Moderate, lean conservative (3)
- O Moderate (4)
- O Moderate, lean liberal (5)
- O Liberal (6)
- O Very liberal (7)

End of Block: Demograpics

Start of Block: Debriefing

Debriefing Thank you for participating in this study on moral values and social groups!

In this study, we were interested in whether people with different political beliefs endorse certain moral values more depending on what kind of group the moral value refers to. Some people think that liberals and conservatives have very different values, but we think that this might be because some measures of moral values make reference to specific groups. Our study is looking at this question.

Your participation will help us increase our understanding of the similarities and dissimilarities between liberals and conservatives with regard to their moral value systems and in the future help us develop methods to help people with different worldviews get along.

Please do not share the purpose of the study with other people who might participate in this study. This could make the results of the study less accurate and useful.

Thanks!

Page Break

Code The study is now finished. Thank you for your participation.

To get paid, please go to the next page where you will see your confirmation code. Use this code on the MTurk page where you found the link to the study.

Comments Do you have any comments for us? Please let us know

____

____

____

____

____

End of Block: Debriefing
