# Supplementary material for: The Effect of Ideological Identification on the Endorsement of Moral Values Depends on the Target Group
Source: Pers Soc Psychol Bull. 2018 Oct 13;45(6):851–63. doi: 10.1177/0146167218798822 (PMC6526610; doi:10.1177/0146167218798822)
Supplement: MFC_-_Supplementary_Materials_-_3rd_Revision_-_Final_Version – Supplemental material for The Effect of Ideological Identification on the Endorsement of Moral Values Depends on the Target Group [file MFC_-_Supplementary_Materials_-_3rd_Revision_-_Final_Version.pdf]

Supplementary Materials

Table SM1

MFQ Items with Explicit or Implicit Ideologically-Relevant Target Group as Identified by the Authors

No	Foundation	Item	Target Group
1	Care/Harm	Whether or not someone suffered emotionally	
2	Fairness/Cheating	Whether or not some people were treated differently from others	
3	Loyalty/Betrayal	Whether or not someone's action showed love for his or her country	Blind patriotism is associated with conservatism (Schatz et al., 1999).
4	Authority/Subversion	Whether or not someone showed a lack of respect for authority	The term authority is – without further specification – often understood as conservative authority (Frimer et al., 2014).
5	Sanctity/Degradation	Whether or not someone violated standards of purity and decency	The terms purity and decency might be – without further specification – understood as conservative standards of purity and decency.
6	Care/Harm	Whether or not someone cared for someone weak or vulnerable	
7	Fairness/Cheating	Whether or not someone acted unfairly	
8	Loyalty/Betrayal	Whether or not someone did something to betray his or her group	
9	Authority/Subversion	Whether or not someone conformed to the traditions of society	The term traditions might be – without further specification –understood as conservative traditions.
10	Sanctity/Degradation	Whether or not someone did something disgusting	

11	Care/Harm	Whether or not someone was cruel	
12	Fairness/Cheating	Whether or not someone was denied his or her rights	The term rights might be – without further specification – associated with liberal target groups.
13	Loyalty/Betrayal	Whether or not someone showed a lack of loyalty	
14	Authority/Subversion	Whether or not an action caused chaos or disorder	
15	Sanctity/Degradation	Whether or not someone acted in a way that God would approve of	Conservatives are, on average, more likely to believe in god (Pew Research Center, 2014).
16	Care/Harm	Compassion for those who are suffering is the most crucial virtue	
17	Fairness/Cheating	When the government makes laws, the number one principle should be ensuring that everyone is treated fairly	
18	Loyalty/Betrayal	I am proud of my country's history	Blind patriotism is associated with conservatism (Schatz et al., 1999).
19	Authority/Subversion	Respect for authority is something all children need to learn	The term authority is – without further specification – often understood as conservative authority (Frimer et al., 2014).
20	Sanctity/Degradation	People should not do things that are disgusting, even if no one is harmed	
21	Care/Harm	One of the worst things a person could do is hurt a defenseless animal	Vegetarianism and veganism are associated with progressive beliefs (Koch et al., 2016).
22	Fairness/Cheating	Justice is the most important requirement for a society	
23	Loyalty/Betrayal	People should be loyal to their family members, even when they have done something wrong	Families are associated with conservatism (Koch et al., 2016).
24	Authority/Subversion	Men and women each have different roles to play in society	Men are more associated with conservatism

			whereas women are more associated with liberalism (Koch et al., 2016).
25	Sanctity/Degradation	I would call some acts wrong on the grounds that they are unnatural	The term unnatural acts might be – without further specification – understood as less common sexual behaviors (e.g. homosexuality).
26	Care/Harm	It can never be right to kill a human being	
27	Fairness/Cheating	I think it's morally wrong that rich children inherit a lot of money while poor children inherit nothing	Rich people are more associated with conservatism whereas poor people are more associated with liberalism (Koch et al., 2016).
28	Loyalty/Betrayal	It is more important to be a team player than to express oneself	
29	Authority/Subversion	If I were a soldier and disagreed with my commanding officer's orders, I would obey anyway because that is my duty	The military is associated with conservatism (Feinberg & Willer, 2015).
30	Sanctity/Degradation	Chastity is an important and valuable virtue	Chastity is a virtue which importance is often derived from religious scriptures (cf. the Christian seven virtues, for example) and conservatives are, on average, more likely to believe in god (Pew Research Center, 2014).

Note: The items we highlight are not necessarily irrelevant measures of moral foundations. The issue is that certain target groups are overrepresented while other target groups are not referenced at all. For instance, patriotism may be a valid indicator of loyalty values, but that is only one type of loyalty value and loyalty may emerge in other ways for liberal participants.

Tables for Results of Multiple Regression Analyses for Studies 1 and 2

Table SM2.1: *The Unstandardized and Standardized Regression Coefficients and Standard Errors for the Effects of Experimental Condition (Dummy Coded), Ideological Identification, and Their Interactions on Endorsement of the Care/Harm Foundation in Study 1*

	Model 1: Orig. Condition as Ref. Category		Model 2: Lib. Condition as Ref. Category		Model 3: Con. Condition as Ref. Category		Model 4: Mod. Condition as Ref. Category	
	b (SE)	*β*	*b (SE)*	*β*	*b (SE)*	*β*	*b (SE)*	*β*
Intercept	3.57	-	3.01	-	3.07	-	2.96	-
	(0.09)	***	(0.09)	***	(0.09)	***	(0.09)	***
Original Condition	Ref. Category		0.55	0.24	0.49	0.21	0.61	0.26
			(0.12)	***	(0.12)	***	(0.12)	***
Liberal Condition	-0.55	-0.23	Ref. Category		-0.06	-0.03	0.05	0.02
	(0.12)	***			(0.12)		(0.13)	
Conservative Condition	-0.49	-0.21	0.06	0.03	Ref. Category		0.11	0.05
	(0.12)	***	(0.12)				(0.13)	
Moderate Condition	-0.61	-0.25	-0.05	-0.02	-0.11	-0.05	Ref. Category	
	(0.12)	***	(0.13)		(0.13)			
Ideological Identification (II)	-0.02	-0.03	-0.10	-0.18	-0.02	-0.03	-0.09	-0.16
	(0.05)		(0.05)	*	(0.05)		(0.05)	+
Original Condition × II	Ref. Category		0.09	0.07	0.00	0.00	0.07	0.06
			(0.07)		(0.07)		(0.07)	
Liberal Condition × II	-0.09	-0.07	Ref. Category		-0.08	-0.07	-0.01	-0.01
	(0.07)				(0.07)		(0.07)	
Conserva. Condition × II	-0.00	-0.00	0.08	0.07	Ref. Category		0.07	0.06
	(0.07)		(0.07)				(0.07)	
Moderate Condition × II	-0.07	-0.06	0.01	0.01	-0.07	-0.06	Ref. Category	
	(0.07)		(0.07)		(0.07)			
Adjusted R^2	0.05							
Sample size (*n*)	542							

Notes. $^{+}p < .10$, $^{*}p < .05$, $^{**}p < .01$, $^{***}p < .001$. The standardized regression coefficients are taken from a model in which the dependent variable has been standardized by subtracting the mean and then dividing by the standard deviation. The independent variables (ideological identification and the condition dummy variables) were also divided by their standard deviation but the mean was not subtracted. This strategy was applied to maintain meaningful interpretation of the null point for each variable. For ideological identification, 0 means “moderate”. For the dummy variables, this ensures that the reported effect of ideological identification is the effect of ideological identification for the respective reference category.

Table SM2.2: *The Unstandardized and Standardized Regression Coefficients and Standard Errors for the Effects of Experimental Condition (Dummy Coded), Ideological identification, and Their Interactions on Endorsement of the Fairness/Cheating Foundation in Study 1*

	Model 1: Orig. Condition as Ref. Category		Model 2: Lib. Condition as Ref. Category		Model 3: Con. Condition as Ref. Category		Model 4: Mod. Condition as Ref. Category	
	b (SE)	*β*	*b (SE)*	*β*	*b (SE)*	*β*	*b (SE)*	*β*
Intercept	3.57	-	3.02	-	3.08	-	3.24	-
	(0.09)	***	(0.09)	***	(0.09)	***	(0.09)	***
Original Condition	Ref. Category		0.56	0.23	0.50	0.20	0.33	0.14
			(0.13)	***	(0.13)	***	(0.13)	**
Liberal Condition	-0.56	-0.22	Ref. Category		-0.06	-0.02	-0.22	-0.09
	(0.13)	***			(0.13)		(0.13)	+
Conservative Condition	-0.50	-0.20	0.06	0.02	Ref. Category		-0.16	-0.07
	(0.13)	***	(0.13)				(0.13)	
Moderate Condition	-0.33	-0.13	0.22	0.09	0.16	0.07	Ref. Category	
	(0.13)	**	(0.13)	+	(0.13)			
Ideological Identification (II)	-0.10	-0.17	-0.23	-0.40	-0.04	-0.06	-0.12	-0.21
	(0.05)	*	(0.05)	***	(0.05)		(0.05)	*
Original Condition × II	Ref. Category		0.13	0.10	-0.06	-0.05	0.02	0.02
			(0.07)	+	(0.07)		(0.07)	
Liberal Condition × II	-0.13	-0.10	Ref. Category		-0.20	-0.14	-0.11	-0.08
	(0.07)	+			(0.07)	**	(0.07)	
Conserva. Condition × II	0.06	0.05	0.20	0.15	Ref. Category		0.09	0.06
	(0.07)		(0.07)	**			(0.07)	
Moderate Condition × II	-0.02	-0.01	0.11	0.08	-0.09	-0.06	Ref. Category	
	(0.07)		(0.07)		(0.07)			
Adjusted R^2	0.08							
Sample size (*n*)	542							

Notes. $^{+}p < .10$, $^{*}p < .05$, $^{**}p < .01$, $^{***}p < .001$. The standardized regression coefficients are taken from a model in which the dependent variable has been standardized by subtracting the mean and then dividing by the standard deviation. The independent variables (ideological identification and the condition dummy variables) were also divided by their standard deviation but the mean was not subtracted. This strategy was applied to maintain meaningful interpretation of the null point for each variable. For ideological identification, 0 means “moderate”. For the dummy variables, this ensures that the reported effect of ideological identification is the effect of ideological identification for the respective reference category.

Table SM2.3: *The Unstandardized and Standardized Regression Coefficients and Standard Errors for the Effects of Experimental Condition (Dummy Coded), Ideological Identification, and Their Interactions on Endorsement of the Loyalty/Betrayal Foundation in Study 1*

	Model 1: Orig. Condition as Ref. Category		Model 2: Lib. Condition as Ref. Category		Model 3: Con. Condition as Ref. Category		Model 4: Mod. Condition as Ref. Category	
	b (SE)	*β*	*b (SE)*	*β*	*b (SE)*	*β*	*b (SE)*	*β*
Intercept	2.44	-	1.79	-	1.75	-	2.12	-
	(0.08)	***	(0.08)	***	(0.08)	***	(0.09)	***
Original Condition	Ref. Category		0.64	0.27	0.68	0.29	0.32	0.13
			(0.12)	***	(0.12)	***	(0.12)	**
Liberal Condition	-0.64	-0.27	Ref. Category		0.04	0.02	-0.33	-0.13
	(0.12)	***			(0.12)		(0.12)	**
Conservative Condition	-0.68	-0.29	-0.04	-0.02	Ref. Category		-0.36	-0.15
	(0.12)	***	(0.12)				(0.12)	**
Moderate Condition	-0.32	-0.13	0.33	0.13	0.36	0.15	Ref. Category	
	(0.12)	**	(0.12)	**	(0.12)	**		
Ideological Identification	0.22	0.38	-0.10	-0.18	0.26	0.46	0.12	0.20
(II)	(0.05)	***	(0.04)	*	(0.04)	***	(0.04)	**
Original Condition × II	Ref. Category		0.32	0.24	-0.04	-0.03	0.10	0.08
			(0.06)	***	(0.06)		(0.06)	
Liberal Condition × II	-0.32	-0.24	Ref. Category		-0.36	-0.27	-0.22	-0.16
	(0.06)	***			(0.06)	***	(0.06)	***
Conserva. Condition × II	0.04	0.03	0.36	0.28	Ref. Category		0.14	0.11
	(0.06)		(0.06)	***			(0.06)	*
Moderate Condition × II	-0.10	-0.08	0.22	0.16	-0.14	-0.11	Ref. Category	
	(0.06)		(0.06)	***	(0.06)	*		
Adjusted R^2				0.17				
Sample size (*n*)				542				

Notes. $^+p < .10$, $^*p < .05$, $^{**}p < .01$, $^{***}p < .001$. The standardized regression coefficients are taken from a model in which the dependent variable has been standardized by subtracting the mean and then dividing by the standard deviation. The independent variables (ideological identification and the condition dummy variables) were also divided by their standard deviation but the mean was not subtracted. This strategy was applied to maintain meaningful interpretation of the null point for each variable. For ideological identification, 0 means "moderate". For the dummy variables, this ensures that the reported effect of ideological identification is the effect of ideological identification for the respective reference category.

Table SM2.4: *The Unstandardized and Standardized Regression Coefficients and Standard Errors for the Effects of Experimental Condition (Dummy Coded), Ideological Identification, and Their Interactions on Endorsement of the Authority/Subversion Foundation in Study 1*

	Model 1: Orig. Condition as Ref. Category		Model 2: Lib. Condition as Ref. Category		Model 3: Con. Condition as Ref. Category		Model 4: Mod. Condition as Ref. Category	
	b (SE)	*β*	*b (SE)*	*β*	*b (SE)*	*β*	*b (SE)*	*β*
Intercept	2.74	-	2.04	-	2.04	-	2.33	-
	(0.09)	***	(0.09)	***	(0.09)	***	(0.09)	***
Original Condition	Ref. Category		0.70	0.27	0.70	0.27	0.41	0.16
			(0.12)	***	(0.12)	***	(0.12)	***
Liberal Condition	-0.70	-0.27	Ref. Category		-0.00	-0.00	-0.29	-0.11
	(0.12)	***			(0.12)		(0.13)	*
Conservative Condition	-0.70	-0.27	0.00	0.00	Ref. Category		-0.29	-0.11
	(0.12)	***	(0.12)				(0.13)	*
Moderate Condition	-0.41	-0.16	0.29	0.11	0.29	0.11	Ref. Category	
	(0.12)	***	(0.13)	*	(0.13)	*		
Ideological Identification	0.27	0.44	-0.06	-0.09	0.32	0.52	0.16	0.27
(II)	(0.05)	***	(0.05)		(0.05)	***	(0.05)	***
Original Condition × II	Ref. Category		0.33	0.23	-0.05	-0.03	0.11	0.08
			(0.07)	***	(0.07)		(0.07)	
Liberal Condition × II	-0.33	-0.23	Ref. Category		-0.37	-0.26	-0.22	-0.16
	(0.07)	***			(0.07)	***	(0.07)	***
Conserva. Condition × II	0.05	0.03	0.37	0.27	Ref. Category		0.15	0.11
	(0.07)		(0.07)	***			(0.07)	*
Moderate Condition × II	-0.11	-0.07	0.22	0.15	-0.15	-0.11	Ref. Category	
	(0.07)		(0.07)	***	(0.07)	*		
Adjusted R^2	0.20							
Sample size (*n*)	542							

Notes. ^+p < .10, *p < .05, **p < .01, ***p < .001. The standardized regression coefficients are taken from a model in which the dependent variable has been standardized by subtracting the mean and then dividing by the standard deviation. The independent variables (ideological identification and the condition dummy variables) were also divided by their standard deviation but the mean was not subtracted. This strategy was applied to maintain meaningful interpretation of the null point for each variable. For ideological identification, 0 means "moderate". For the dummy variables, this ensures that the reported effect of ideological identification is the effect of ideological identification for the respective reference category.

Table SM2.5: *The Unstandardized and Standardized Regression Coefficients and Standard Errors for the Effects of Experimental Condition (Dummy Coded), Ideological Identification, and Their Interactions on Endorsement of the Sanctity/Degradation Foundation in Study 1*

	Model 1: Orig. Condition as Ref. Category		Model 2: Lib. Condition as Ref. Category		Model 3: Con. Condition as Ref. Category		Model 4: Mod. Condition as Ref. Category	
	b (SE)	*β*	*b (SE)*	*β*	*b (SE)*	*β*	*b (SE)*	*β*
Intercept	2.27	-	1.83	-	1.69	-	2.02	-
	(0.10)	***	(0.10)	***	(0.10)	***	(0.10)	***
Original Condition	Ref. Category		0.45	0.16	0.58	0.20	0.25	0.09
			(0.14)	**	(0.14)	***	(0.14)	+
Liberal Condition	-0.45	-0.15	Ref. Category		0.13	0.05	-0.20	-0.07
	(0.14)	**			(0.14)		(0.14)	
Conservative Condition	-0.58	-0.20	-0.13	-0.05	Ref. Category		-0.33	-0.12
	(0.14)	***	(0.14)				(0.14)	*
Moderate Condition	-0.25	-0.09	0.20	0.07	0.33	0.11	Ref. Category	
	(0.14)	+	(0.14)		(0.14)	*		
Ideological Identification	0.34	0.50	0.05	0.08	0.30	0.44	0.26	0.38
(II)	(0.05)	***	(0.05)		(0.05)	***	(0.05)	***
Original Condition × II	Ref. Category		0.29	0.18	0.04	0.03	0.08	0.05
			(0.08)	***	(0.08)		(0.08)	
Liberal Condition × II	-0.29	-0.18	Ref. Category		-0.25	-0.16	-0.21	-0.13
	(0.08)	***			(0.08)	**	(0.08)	**
Conserva. Condition × II	-0.04	-0.03	0.25	0.16	Ref. Category		0.04	0.02
	(0.08)		(0.08)	**			(0.08)	
Moderate Condition × II	-0.08	-0.05	0.21	0.13	-0.04	-0.02	Ref. Category	
	(0.08)		(0.08)	**	(0.08)			
Adjusted R^2				0.17				
Sample size (*n*)				542				

Notes. $^+p < .10$, $*p < .05$, $**p < .01$, $***p < .001$. The standardized regression coefficients are taken from a model in which the dependent variable has been standardized by subtracting the mean and then dividing by the standard deviation. The independent variables (ideological identification and the condition dummy variables) were also divided by their standard deviation but the mean was not subtracted. This strategy was applied to maintain meaningful interpretation of the null point for each variable. For ideological identification, 0 means "moderate". For the dummy variables, this ensures that the reported effect of ideological identification is the effect of ideological identification for the respective reference category.

Table SM3: *The Means and Standard Deviations of the Endorsement of the Five Moral Foundation in Each Experimental Condition in Study 1*

	Original Condition		Liberal Condition		Conservative Condition		Moderate Condition		Total	
Moral Foundation	*M*	*SD*	*M*	*SD*	*M*	*SD*	*M*	*SD*	*M*	*SD*
Care/Harm	3.57	0.80	3.06	1.05	3.08	1.14	3.00	0.98	3.18	1.02
Fairness/Cheating	3.59	0.79	3.12	1.16	3.10	1.22	3.29	1.00	3.28	1.07
Loyalty/Betrayal	2.40	0.97	1.84	0.98	1.63	1.10	2.07	0.97	1.98	1.04
Auth./Subversion	2.69	1.00	2.06	1.04	1.89	1.17	2.26	1.10	2.22	1.12
Sanc./Degradation	2.21	1.36	1.80	1.13	1.55	1.21	1.92	1.20	1.87	1.25
Sample size (*n*)	138		135		139		130		542	

Table SM4.1: *The Unstandardized Regression Coefficients and Standard Errors for the Effects of Experimental Condition (Dummy Coded), Ideological Identification, and Their Interactions on Endorsement of the Care/Harm Foundation in Study 2*

	Model 1: Orig. Condition as Ref. Category		Model 2: Lib. Condition as Ref. Category		Model 3: Con. Condition as Ref. Category	
	b (SE)	*β*	*b (SE)*	*β*	*b (SE)*	*β*
Intercept	3.50	-	2.62	-	2.62	-
	(0.07)	***	(0.07)	***	(0.07)	***
Original Condition	Ref. Category		0.88	0.47	0.88	0.47
			(0.10)	***	(0.10)	***
Liberal Condition	-0.88	-0.47	Ref. Category		-0.00	-0.00
	(0.10)	***			(0.10)	
Conservative Condition	-0.88	-0.47	0.00	0.00	Ref. Category	
	(0.10)	***	(0.10)			
Ideological Identification (II)	0.03	0.07	-0.08	-0.15	0.16	0.31
	(0.04)		(0.04)	*	(0.04)	***
Original Condition × II	Ref. Category		0.11	0.10	-0.12	-0.12
			(0.05)	*	(0.05)	*
Liberal Condition × II	-0.11	-0.10	Ref. Category		-0.24	-0.22
	(0.05)	*			(0.05)	***
Conservative Condition × II	0.12	0.12	0.24	0.22	Ref. Category	
	(0.05)	*	(0.05)	***		
Adjusted R^2			0.27			
Sample size (*n*)			416			

Notes. $^{+}p < .10$, $^{*}p < .05$, $^{**}p < .01$, $^{***}p < .001$. The standardized regression coefficients are taken from a model in which the dependent variable has been standardized by subtracting the mean and then dividing by the standard deviation. The independent variables (ideological identification and the condition dummy variables) were also divided by their standard deviation but the mean was not subtracted. This strategy was applied to maintain meaningful interpretation of the null point for each variable. For ideological identification, 0 means “moderate”. For the dummy variables, this ensures that the reported effect of ideological identification is the effect of ideological identification for the respective reference category.

Table SM4.2: *The Unstandardized and Standardized Regression Coefficients and Standard Errors for the Effects of Experimental Condition (Dummy Coded), Ideological Identification, and Their Interactions on Endorsement of the Fairness/Cheating Foundation in Study 2*

	Model 1: Orig. Condition as Ref. Category		Model 2: Lib. Condition as Ref. Category		Model 3: Con. Condition as Ref. Category	
	b (SE)	*β*	*b (SE)*	*β*	*b (SE)*	*β*
Intercept	3.96	-	3.95	-	3.99	-
	(0.05)	***	(0.06)	***	(0.06)	***
Original Condition	Ref. Category		0.01	0.01	-0.03	-0.02
			(0.08)		(0.08)	
Liberal Condition	-0.01	-0.01	Ref. Category		-0.04	-0.03
	(0.08)				(0.08)	
Conservative Condition	0.03	0.02	0.04	0.03	Ref. Category	
	(0.08)		(0.08)			
Ideological Identification (II)	0.07	0.20	-0.04	-0.11	0.06	0.17
	(0.03)	*	(0.03)		(0.03)	+
Original Condition × II	Ref. Category		0.11	0.15	0.01	0.01
			(0.04)	**	(0.04)	
Liberal Condition × II	-0.11	-0.14	Ref. Category		-0.10	-0.13
	(0.04)	**			(0.04)	*
Conservative Condition × II	-0.01	-0.01	0.10	0.13	Ref. Category	
	(0.04)		(0.04)	*		
Adjusted R^2			0.02			
Sample size (*n*)			416			

Notes. $^{+}p < .10$, $^{*}p < .05$, $^{**}p < .01$, $^{***}p < .001$. The standardized regression coefficients are taken from a model in which the dependent variable has been standardized by subtracting the mean and then dividing by the standard deviation. The independent variables (ideological identification and the condition dummy variables) were also divided by their standard deviation but the mean was not subtracted. This strategy was applied to maintain meaningful interpretation of the null point for each variable. For ideological identification, 0 means "moderate". For the dummy variables, this ensures that the reported effect of ideological identification is the effect of ideological identification for the respective reference category.

Table SM4.3: *The Unstandardized and Standardized Regression Coefficients and Standard Errors for the Effects of Experimental Condition (Dummy Coded), Ideological Identification, and Their Interactions on Endorsement of the Loyalty/Betrayal Foundation in Study 2*

	Model 1: Orig. Condition as Ref. Category		Model 2: Lib. Condition as Ref. Category		Model 3: Con. Condition as Ref. Category	
	b (SE)	*β*	*b (SE)*	*β*	*b (SE)*	*β*
Intercept	2.91	-	2.88	-	2.84	-
	(0.07)	***	(0.07)	***	(0.07)	***
Original Condition	Ref. Category		0.03	0.02	0.07	0.04
			(0.10)		(0.10)	
Liberal Condition	-0.03	-0.02	Ref. Category		0.04	0.02
	(0.10)				(0.10)	
Conservative Condition	-0.07	-0.04	-0.04	-0.02	Ref. Category	
	(0.10)		(0.10)			
Ideological Identification (II)	0.26	0.50	0.04	0.08	0.26	0.52
	(0.04)	***	(0.04)		(0.04)	***
Original Condition × II	Ref. Category		0.22	0.20	-0.01	-0.01
			(0.05)	***	(0.06)	
Liberal Condition × II	-0.22	-0.20	Ref. Category		-0.23	-0.21
	(0.05)	***			(0.06)	***
Conservative Condition × II	0.01	0.01	0.23	0.21	Ref. Category	
	(0.06)		(0.06)	***		
Adjusted R^2			0.16			
Sample size (*n*)			416			

Notes. $^+p < .10$, $*p < .05$, $**p < .01$, $***p < .001$. The standardized regression coefficients are taken from a model in which the dependent variable has been standardized by subtracting the mean and then dividing by the standard deviation. The independent variables (ideological identification and the condition dummy variables) were also divided by their standard deviation but the mean was not subtracted. This strategy was applied to maintain meaningful interpretation of the null point for each variable. For ideological identification, 0 means “moderate”. For the dummy variables, this ensures that the reported effect of ideological identification is the effect of ideological identification for the respective reference category.

Table SM4.4: *The Unstandardized and Standardized Regression Coefficients and Standard Errors for the Effects of Experimental Condition (Dummy Coded), Ideological Identification, and Their Interactions on Endorsement of the Authority/Subversion Foundation in Study 2*

	Model 1: Orig. Condition as Ref. Category		Model 2: Lib. Condition as Ref. Category		Model 3: Con. Condition as Ref. Category	
	b (SE)	*β*	*b (SE)*	*β*	*b (SE)*	*β*
Intercept	3.23	-	3.09	-	3.14	-
	(0.06)	***	(0.07)	***	(0.07)	***
Original Condition	Ref. Category		0.14	0.09	0.09	0.06
			(0.09)		(0.09)	
Liberal Condition	-0.14	-0.09	Ref. Category		-0.05	-0.03
	(0.09)				(0.09)	
Conservative Condition	-0.09	-0.06	0.05	0.03	Ref. Category	
	(0.09)		(0.09)			
Ideological Identification (II)	0.16	0.36	-0.05	-0.11	0.20	0.46
	(0.04)	***	(0.03)		(0.04)	***
Original Condition × II	Ref. Category		0.21	0.23	-0.04	-0.05
			(0.05)	***	(0.05)	
Liberal Condition × II	-0.21	-0.22	Ref. Category		-0.25	-0.27
	(0.05)	***			(0.05)	***
Conservative Condition × II	0.04	0.05	0.25	0.27	Ref. Category	
	(0.05)		(0.05)	***		
Adjusted R^2			0.11			
Sample size (*n*)			416			

Notes. $^{+}p < .10$, $*p < .05$, $**p < .01$, $***p < .001$. The standardized regression coefficients are taken from a model in which the dependent variable has been standardized by subtracting the mean and then dividing by the standard deviation. The independent variables (ideological identification and the condition dummy variables) were also divided by their standard deviation but the mean was not subtracted. This strategy was applied to maintain meaningful interpretation of the null point for each variable. For ideological identification, 0 means "moderate". For the dummy variables, this ensures that the reported effect of ideological identification is the effect of ideological identification for the respective reference category.

Table SM4.5: *The Unstandardized and Standardized Regression Coefficients and Standard Errors for the Effects of Experimental Condition (Dummy Coded), Ideological Identification, and Their Interactions on Endorsement of the Sanctity/Degradation Foundation in Study 2*

	Model 1: Orig. Condition as Ref. Category		Model 2: Lib. Condition as Ref. Category		Model 3: Con. Condition as Ref. Category	
	b (SE)	*β*	*b (SE)*	*β*	*b (SE)*	*β*
Intercept	3.55	-	3.72	-	3.78	-
	(0.08)	***	(0.09)	***	(0.09)	***
Original Condition	Ref. Category		-0.17	-0.08	-0.24	-0.11
			(0.12)		(0.12)	*
Liberal Condition	0.17	0.08	Ref. Category		-0.07	-0.03
	(0.12)				(0.12)	
Conservative Condition	0.24	0.11	0.07	0.03	Ref. Category	
	(0.12)	*	(0.12)			
Ideological Identification (II)	0.23	0.40	0.13	0.22	0.24	0.41
	(0.05)	***	(0.04)	**	(0.05)	***
Original Condition × II	Ref. Category		0.11	0.09	-0.00	-0.00
			(0.06)	+	(0.07)	
Liberal Condition × II	-0.11	-0.09	Ref. Category		-0.11	-0.09
	(0.06)	+			(0.07)	+
Conservative Condition × II	0.00	0.00	0.11	0.09	Ref. Category	
	(0.07)		(0.07)	+		
Adjusted R^2			0.12			
Sample size (*n*)			416			

Notes. $^{+}p < .10$, $*p < .05$, $**p < .01$, $***p < .001$. The standardized regression coefficients are taken from a model in which the dependent variable has been standardized by subtracting the mean and then dividing by the standard deviation. The independent variables (ideological identification and the condition dummy variables) were also divided by their standard deviation but the mean was not subtracted. This strategy was applied to maintain meaningful interpretation of the null point for each variable. For ideological identification, 0 means “moderate”. For the dummy variables, this ensures that the reported effect of ideological identification is the effect of ideological identification for the respective reference category.

Table SM5: *The Means and Standard Deviations of the Endorsement of the Five Moral Foundation in Each Experimental Condition in Study 2*

	Original Condition		Liberal Condition		Conservative Condition		Total	
Moral Foundation	*M*	*SD*	*M*	*SD*	*M*	*SD*	*M*	*SD*
Care/Harm	3.49	0.72	2.67	0.81	2.51	0.79	2.89	0.88
Fairness/Cheating	3.93	0.56	3.97	0.66	3.94	0.62	3.95	0.61
Loyalty/Betrayal	2.78	0.92	2.85	0.82	2.65	0.87	2.76	0.87
Auth./Subversion	3.15	0.76	3.12	0.73	2.99	0.78	3.09	0.76
Sanc./Degradation	3.42	1.02	3.64	0.96	3.61	1.00	3.56	1.00
Sample size (*n*)	141		134		141		416	

Main Effects of Target Conditions

Statistical Approach

We tested for main effects of target condition in a model without interaction effects. That is, the endorsement of each moral foundation was the dependent variable and ideological identification and target condition (dummy coded) were the predictors.

Results – Study 1

For the care/harm foundation, participants in the original condition scored significantly higher than participants in the three other conditions, all bs > 0.50, all SEs = 0.12, all $t(537)$s > 4.21, all ps < .001. The other target condition main effects were non-significant, all ps > .509.

For the fairness/cheating foundation, participants in the original condition scored significantly higher than participants in the three other conditions, all bs > 0.32, all SEs < 0.13, all $t(537)$s > 2.60, all ps < .010. The other target condition main effects were non-significant, all ps > .101.

For the loyalty/betrayal foundation, participants in the original condition scored significantly higher than participants in the three other conditions, all bs > 0.29, all SEs = 0.12, all $t(537)$s > 2.48, all ps < .014. Participants in the moderate target condition scored significantly higher than participants in the conservative target condition, $b = 0.43$, $SE = 0.12$, $t(537) = 3.64$, $p < .001$, and marginally significantly higher than participants in the liberal target condition, $b = 0.23$, $SE = 0.12$, $t(537) = 1.91$, $p = .057$. Participants in the liberal target condition scored marginally significantly higher than participants in the conservative target condition, $b = 0.20$, $SE = 0.12$, $t(537) = 1.73$, $p = .084$.

For the authority/subversion foundation, participants in the original condition scored significantly higher than participants in the three other conditions, all bs > 0.39, all SEs < 0.13, all $t(537)$s > 3.09, all ps < .003. Participants in the moderate target condition scored significantly

higher than participants in the conservative target condition, $b = 0.36$, $SE = 0.13$, $t(537) = 2.85$, $p = .004$. The other target condition main effects were non-significant, both *p*s > .127.

For the sanctity/degradation foundation, participants in the original condition scored significantly higher than participants in the liberal and conservative target conditions, both *b*s > 0.34, both *SE*s = 0.14, both *t*(537)s > 2.49, both *p*s < .014, and marginally significantly higher than participants in the moderate target condition, $b = 0.24$, $SE = 0.14$, $t(537) = 1.71$, $p = .089$. Participants in the moderate target condition scored significantly higher than participants in the conservative target condition, $b = 0.35$, $SE = 0.14$, $t(537) = 2.51$, $p = .012$. Participants in the liberal target condition scored marginally significantly higher than participants in the conservative target condition, $b = 0.24$, $SE = 0.14$, $t(537) = 1.76$, $p = .079$. Participants in the moderate and liberal target conditions did not differ significantly from each other, $p = .448$.

Results – Study 2

For the care/harm foundation, participants in the original condition scored significantly higher than participants in the liberal and conservative target conditions, both *b*s > 0.81, both *SE*s = 0.09, both *t*(412)s > 8.69, both *p*s < .001. Participants in the liberal target condition scored marginally significantly higher than participants in the conservative target condition, $b = 0.16$, $SE = 0.09$, $t(412) = 1.70$, $p = .089$.

For the fairness/cheating foundation, all target condition main effects were non-significant, all *p*s > .496.

For the loyalty/betrayal foundation, participants in the liberal target condition scored marginally significantly higher than participants in the conservative target condition, $b = 0.19$, $SE = 0.10$, $t(412) = 1.91$, $p = .057$. The other target condition main effects were non-significant, both *p*s > .336.

For the authority/subversion foundation, all target condition main effects were non-significant, all *p*s $> .113$.

For the sanctity/degradation foundation, participants in the original condition scored significantly lower than participants in the liberal and conservative target conditions, both *b*s < -0.22, both *SE*s $= 0.11$, both $t(412)$s < -2.03, both *p*s $< .043$. Participants in the liberal and conservative target conditions did not differ significantly from each other, $p = .939$.

Robustness Checks

Controlling for Gender and Age

Motivation and statistical approach. We carried out a robustness check to test if the inclusion of gender and age as covariates affected our conclusions. To test this, we added gender (dummy-coded) and age (mean-centered) to the model for each moral foundation.

Results – Study 1. We found the same results except for the following differences. The difference in the effect of ideological identification on the endorsement of the care/harm foundation between the liberal target condition and the conservative target condition changed from non-significant (b = -0.08, SE = 0.07, $t(534)$ = -1.26, p = .207) to marginally significant (b = -0.11, SE = 0.07, $t(532)$ = -1.72, p = .086). The difference in the effect of ideological identification on the endorsement of the fairness/cheating foundation between the liberal target condition and the moderate target condition changed from non-significant (b = -0.11, SE = 0.07, $t(534)$ = -1.63, p = .104) to marginally significant (b = -0.13, SE = 0.07, $t(532)$ = -1.92, p = .056).

Results – Study 2. We found the same results except for the following differences. The simple effects of ideological identification on the endorsement of the fairness/cheating foundation in the original condition and in the conservative target condition changed from significant (original condition: b = 0.07, SE = 0.03, $t(410)$ = 2.31, p = .022) and marginally significant (conservative target condition: b = 0.06, SE = 0.03, $t(410)$ = 1.95, p = .052) to non-significant (original target condition: b = 0.05, SE = 0.03, $t(408)$ = 1.59, p = .112; conservative target condition: b = 0.05, SE = 0.03, $t(408)$ = 1.52, p = .130). The differences in the effect of ideological identification on the endorsement of the sanctity/degradation foundation between the liberal target condition and the two other conditions changed from marginally significant (difference between liberal target condition and original condition: b = -0.11, SE = 0.06, $t(410)$ = -1.65, p = .099; difference between liberal target condition and conservative target condition: b =

-0.11, *SE* = 0.07, *t*(410) = -1.68, *p* = .093) to non-significant (difference between liberal target condition and original condition: *b* = -0.10, *SE* = 0.06, *t*(408) = -1.57, *p* = .118; difference between liberal target condition and conservative target condition: *b* = -0.11, *SE* = 0.06, *t*(408) = -1.63, *p* = .104).

Non-Linear Effects of Ideological Identification

Motivation and statistical approach. The motivation for conducting these additional analyses was to test whether the target condition had different effects among liberals and conservatives. A reviewer suggested that it could be that the target condition introduced an aversion of parochialism for one ideological group but not for the other group. The political group conflict hypothesis essentially predicts a sort of moral parochialism for both liberals and conservatives, but if the effects are only due to one side of the political spectrum, then it would threaten the validity of our conclusions. To test if the manipulations differed in their effects for liberals and conservatives we reran all analyses while replacing the ideological identification variable used so far with two different variables: ideological type (-1: liberal, 0: moderate, 1: conservative) and ideological extremity (from 0 (moderate) to 3 (very liberal / conservative). We included main effects of these two variables, their interaction (which recreates the linear variable used in our main analyses) as well as all two- and three-way interaction effects with the target condition dummy variable (0: liberal target condition, 1: conservative target condition). If the target condition introduces a parochialism bias, we would expect to find differences between the conservative target condition and the liberal target condition for one of the two ideological groups but not for the other one.

To test for differences in parochialism among liberals and conservatives, we only need two experimental conditions, the conservative target condition (Study 1 n = 139, Study 2 n = 141) and the liberal target condition (Study 1 n = 135, Study 2 n = 134). The other conditions were not

considered because they did not clearly introduce parochialism for the ideological groups. Notably, the results of these additional analyses should be treated with caution because they were not preregistered and because the additional parameters and smaller sample sizes lead to larger standard errors.

Results – Study 1. We report the results of these additional analyses for both studies in two ways. First, we inspect plots of the endorsement of each moral foundation for strong conservatives (7 on the original 1-7 scale), moderate conservatives (5), moderates (4), moderate liberals (3) and strong liberals (1) estimated by the model with all two-way and three-way interaction effects. This visual inspection (see Figures SM1.1 – SM1.5) does generally not suggest that parochialism (higher endorsement of moral values when then target was a member of the ingroup (versus outgroup)) was stronger or only apparent for one of the ideological groups.

This visual pattern was generally bolstered by the second set of analyses. For this analysis, we removed non-significant effects ($p > .05$) and report the results from the final model. For three of the five foundations, we found similar parochialism effects among liberals and conservatives. For the care/harm foundation, none of the parochialism effects were significant. Notably, this lack of parochialism does also not support the hypothesis that parochialism would be stronger for one ideological group than the other. For the sanctity/degradation foundation, we found a significant effect of parochialism among liberals but not among conservatives (this could be due to the lower number of conservatives which explains the higher standard errors). Detailed results for each foundation are provided below.

For the care/harm foundation, none of the main and interaction effects were significant (all *p*s > .072).

For the fairness/cheating foundation, the ideological type × condition interaction effect was significant, $b = 0.48$, $SE = 0.16$, $t(268) = 3.04$, $p = .003$. Simple effects analyses showed that

liberals scored lower in the conservative target condition than in the liberal target condition, $b = -0.41$, $SE = 0.19$, $t(268) = -2.22$, $p = .027$. For moderates, the difference was not significant, $b = 0.06$, $SE = 0.14$, $t(268) = 0.45$, $p = .652$. Conservatives scored higher in the conservative target condition than in the liberal target condition, $b = 0.54$, $SE = 0.24$, $t(268) = 2.30$, $p = .022$. The ideological type × ideological extremity interaction effect was also significant, $b = -0.22$, $SE = 0.10$, $t(268) = -2.14$, $p = .033$. Simple effects analyses showed that, for liberals, the effect of ideological extremity was positive and significant, $b = 0.32$, $SE = 0.12$, $t(268) = 2.73$, $p = .007$. For conservatives, the effect of ideological extremity was negative but non-significant, $b = -0.13$, $SE = 0.14$, $t(268) = -0.98$, $p = .329$. The other interaction effects were non-significant (both ps > .137).

For the loyalty/betrayal foundation, the ideological type × condition interaction effect was significant, $b = 0.66$, $SE = 0.13$, $t(269) = 4.91$, $p < .001$. Simple effects analyses showed that liberals scored lower in the conservative target condition than in the liberal target condition, $b = -0.74$, $SE = 0.16$, $t(269) = -4.66$, $p < .001$. For moderates, the difference was not significant, $b = -0.08$, $SE = 0.12$, $t(269) = -0.68$, $p = .500$. Conservatives scored higher in the conservative target condition than in the liberal target condition, $b = 0.58$, $SE = 0.20$, $t(269) = 2.87$, $p = .004$. The other interaction effects were non-significant (all ps > .050). However, the main effect of ideological extremity was negative and significant, $b = -0.17$, $SE = 0.06$, $t(269) = -2.89$, $p = .004$, indicating that people who identified more strongly as either liberal or conservative scored on average lower in these two conditions.

For the authority/subversion foundation, the ideological type × condition interaction effect was significant, $b = 0.71$, $SE = 0.14$, $t(269) = 5.03$, $p < .001$. Simple effects analyses showed that liberals scored lower in the conservative target condition than in the liberal target condition, $b = -0.75$, $SE = 0.17$, $t(269) = -4.47$, $p < .001$. For moderates, the difference was not

significant, b = -0.04, SE = 0.13, $t(269)$ = -0.29, p = .772. Conservatives scored higher in the conservative target condition than in the liberal target condition, b = 0.67, SE = 0.21, $t(269)$ = 3.19, p = .002. The other interaction effects were non-significant (all ps > .057). However, the main effect of ideological extremity was negative and significant, b = -0.19, SE = 0.06, $t(269)$ = -3.09, p = .002, indicating that people who identified more strongly as either liberal or conservative scored on average lower in these two conditions.

For the sanctity/degradation foundation, the ideological type × condition interaction effect was significant, b = 0.46, SE = 0.15, $t(268)$ = 3.02, p = .003. Simple effects analyses showed that liberals scored lower in the conservative target condition than in the liberal target condition, b = -0.61, SE = 0.18, $t(268)$ = -3.36, p < .001. For moderates, the difference was not significant, b = -0.15, SE = 0.14, $t(268)$ = -1.05, p = .295. Conservatives scored higher in the conservative target condition than in the liberal target condition but this difference was not significant, b = 0.32, SE = 0.23, $t(268)$ = 1.38, p = .169. The ideological type × ideological extremity interaction effect was also significant, b = 0.29, SE = 0.10, $t(268)$ = 2.90, p = .004. Simple effects analyses showed that, for liberals, the effect of ideological extremity was negative and significant, b = -0.38, SE = 0.11, $t(268)$ = -3.36, p < .001. For conservatives, the effect of ideological extremity was positive but non-significant, b = 0.21, SE = 0.13, $t(268)$ = 1.60, p = .111. The other interaction effects were non-significant (both ps > .206).

Results – Study 2. Visual inspection of Figures SM2.1 – SM2.5 generally does not suggest that parochialism (higher endorsement of moral values when then target was a member of the ingroup (versus outgroup)) was stronger or only apparent for one of the ideological groups. This visual pattern was generally bolstered by the second set of analyses. For this analysis, we remove non-significant effects (p > .05) and report the results from the final model. For two of the five foundations, we found similar parochialism effects among liberals and conservatives. For

the fairness/cheating foundation, none of the parochialism effects were significant. Notably, this lack of parochialism does also not support the hypothesis that parochialism would be stronger for one ideological group than the other. For the authority/subversion foundation, we found parochialism effects for both strong liberals and strong conservatives. However, the effect size of the parochialism effect was larger among conservatives. For the sanctity/degradation foundation, we found a significant effect of parochialism among conservatives but not among liberals. Importantly, this should be treated with caution due to the small number of strong conservatives in our sample. Detailed results for each foundation are provided below.

For the care/harm foundation, the ideological type × condition interaction effect was significant, $b = 0.45$, $SE = 0.11$, $t(271) = 4.05$, $p < .001$. Simple effects analyses showed that liberals scored lower in the conservative target condition than in the liberal target condition, $b = -0.47$, $SE = 0.12$, $t(271) = -3.90$, $p < .001$. For moderates, the difference was not significant, $b = -0.03$, $SE = 0.10$, $t(271) = -0.25$, $p = .802$. Conservatives scored higher in the conservative target condition than in the liberal target condition, $b = 0.42$, $SE = 0.17$, $t(271) = 2.45$, $p = .015$. The other interaction effects and the main effect of ideological extremity were non-significant (all *p*s > .116).

For the fairness/cheating foundation, the ideological type × condition interaction effect was significant, $b = 0.20$, $SE = 0.09$, $t(271) = 2.18$, $p = .030$. Simple effects analyses showed that liberals scored lower in the conservative target condition than in the liberal target condition but the effect was only marginally significant, $b = -0.17$, $SE = 0.10$, $t(271) = -1.68$, $p = .095$. For moderates, the difference was not significant, $b = 0.03$, $SE = 0.08$, $t(271) = 0.37$, $p = .708$. Conservatives scored higher in the conservative target condition than in the liberal target condition but the difference was non-significant, $b = 0.23$, $SE = 0.14$, $t(271) = 1.61$, $p = .108$. The

other interaction effects and the main effect of ideological extremity were non-significant (all *p*s > .061).

For the loyalty/betrayal foundation, the ideological type × condition interaction effect was significant, $b = 0.45$, $SE = 0.12$, $t(269) = 3.90$, $p < .001$. Simple effects analyses showed that liberals scored lower in the conservative target condition than in the liberal target condition, $b = -0.50$, $SE = 0.12$, $t(269) = -4.03$, $p < .001$. For moderates, the difference was not significant, $b = -0.04$, $SE = 0.11$, $t(269) = -0.41$, $p = .680$. Conservatives scored higher in the conservative target condition than in the liberal target condition, $b = 0.41$, $SE = 0.18$, $t(269) = 2.22$, $p = .027$. The ideological type x ideological extremity interaction effect was also significant, $b = 0.22$, $SE = 0.08$, $t(269) = 2.77$, $p = .006$. Simple effects analyses showed that, for liberals, the effect of ideological extremity was negative and significant, $b = -0.23$, $SE = 0.08$, $t(269) = -2.98$, $p = .003$. For conservatives, the effect of ideological extremity was positive but marginally significant, $b = 0.21$, $SE = 0.11$, $t(269) = 1.89$, $p = .059$. The other interaction effects were non-significant (both *p*s > .228).

For the authority/subversion foundation, the ideological type × ideological extremity × condition interaction effect was significant, $b = 0.39$, $SE = 0.15$, $t(267) = 2.64$, $p = .009$. Simple effects analyses showed that strong liberals scored lower in the conservative target condition than in the liberal target condition, $b = -0.63$, $SE = 0.19$, $t(267) = -3.33$, $p < .001$. Moderate liberals scored lower in the conservative target condition than in the liberal target condition but this difference was not significant, $b = -0.21$, $SE = 0.18$, $t(267) = -1.18$, $p = .240$. For moderates, the difference was not significant, $b = -0.19$, $SE = 0.17$, $t(267) = -1.11$, $p = .266$. Moderate conservatives scored higher in the conservative target condition than in the liberal target condition but this difference was not significant, $b = 0.19$, $SE = 0.21$, $t(267) = 0.92$, $p = .356$.

Strong conservatives scored higher in the conservative target condition than in the liberal target condition, $b = 1.32$, $SE = 0.35$, $t(267) = 3.83$, $p < .001$.

For the sanctity/degradation foundation, the ideological type × condition interaction effect was significant, $b = 0.31$, $SE = 0.14$, $t(269) = 2.31$, $p = .022$. Simple effects analyses showed that liberals scored lower in the conservative target condition than in the liberal target condition but this difference was not significant, $b = -0.19$, $SE = 0.14$, $t(269) = -1.33$, $p = .184$. For moderates, the difference was also not significant, $b = 0.12$, $SE = 0.12$, $t(269) = 0.98$, $p = .327$. Conservatives scored higher in the conservative target condition than in the liberal target condition, $b = 0.43$, $SE = 0.22$, $t(269) = 2.01$, $p = .045$. The ideological type × ideological extremity interaction effect was also significant, $b = 0.26$, $SE = 0.09$, $t(269) = 2.76$, $p = .006$. Simple effects analyses showed that, for liberals, the effect of ideological extremity was negative and marginally significant, $b = -0.16$, $SE = 0.09$, $t(269) = -1.71$, $p = .089$. For conservatives, the effect of ideological extremity was positive and significant, $b = 0.36$, $SE = 0.13$, $t(269) = 2.76$, $p = .006$. The other interaction effects were non-significant (both *p*s > .721).

Figure SM1.1: *The Regression Intercepts and 95 % Confidence Intervals for Endorsement of the Care/Harm Foundation Depending on Experimental Condition, Ideological Type, and Ideological Extremity in Study 1*

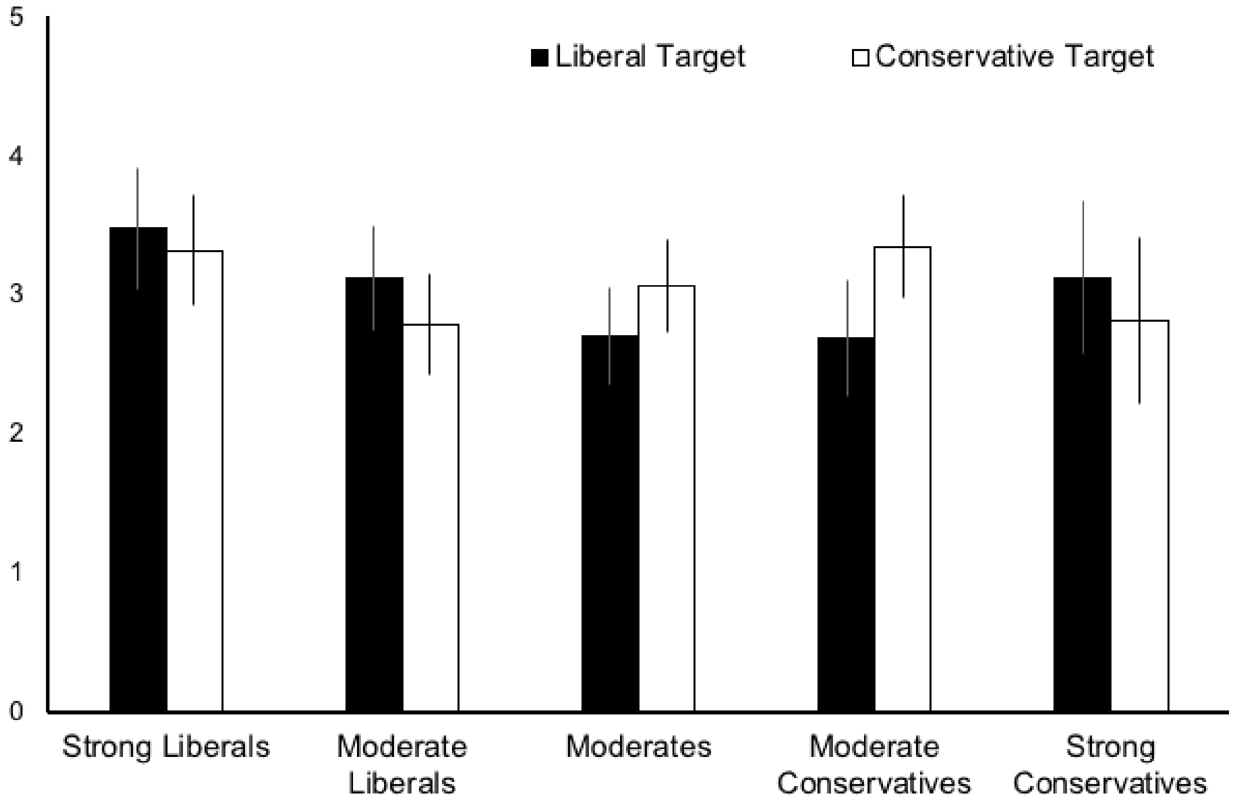

Figure SM1.2: *The Regression Intercepts and 95 % Confidence Intervals for Endorsement of the Fairness/Cheating Foundation Depending on Experimental Condition, Ideological Type, and Ideological Extremity in Study 1*

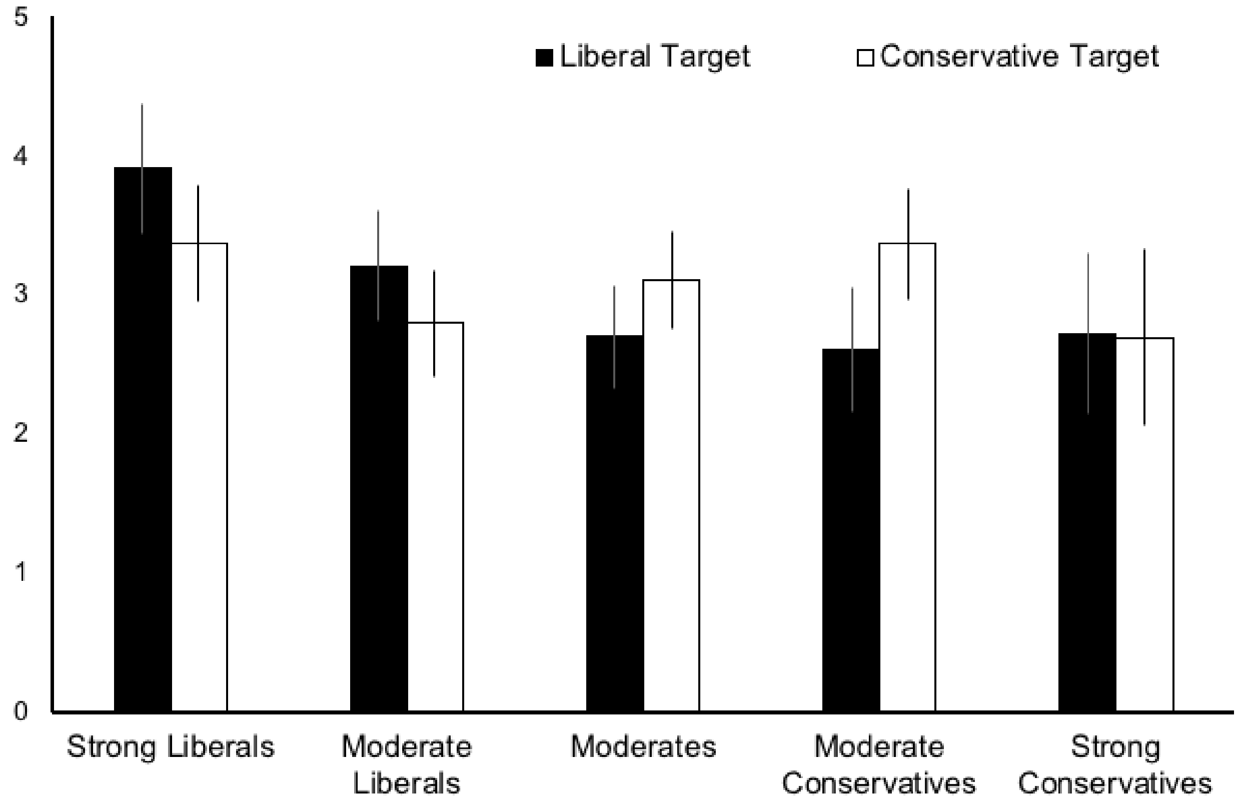

Figure SM1.3: *The Regression Intercepts and 95 % Confidence Intervals for Endorsement of the Loyalty/Betrayal Foundation Depending on Experimental Condition, Ideological Type, and Ideological Extremity in Study 1*

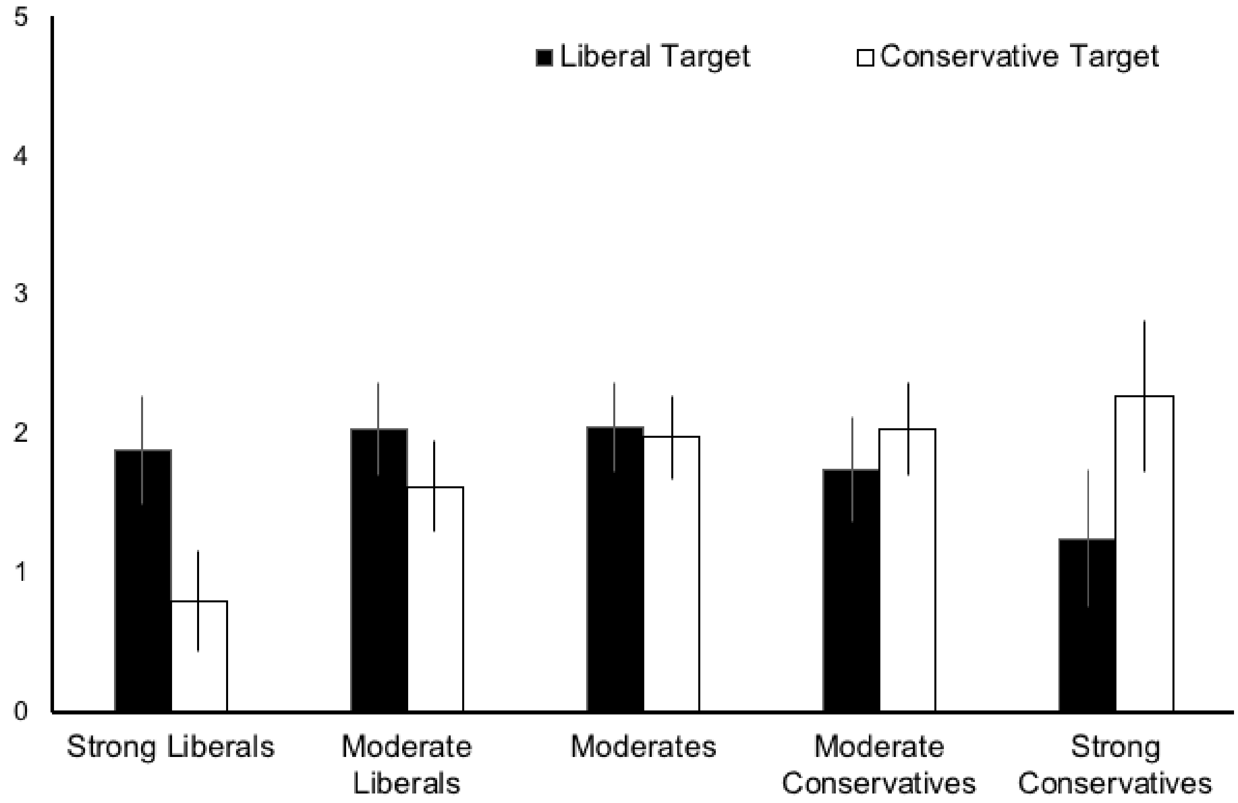

Figure SM1.4: *The Regression Intercepts and 95 % Confidence Intervals for Endorsement of the Authority/Subversion Foundation Depending on Experimental Condition, Ideological Type, and Ideological Extremity in Study 1*

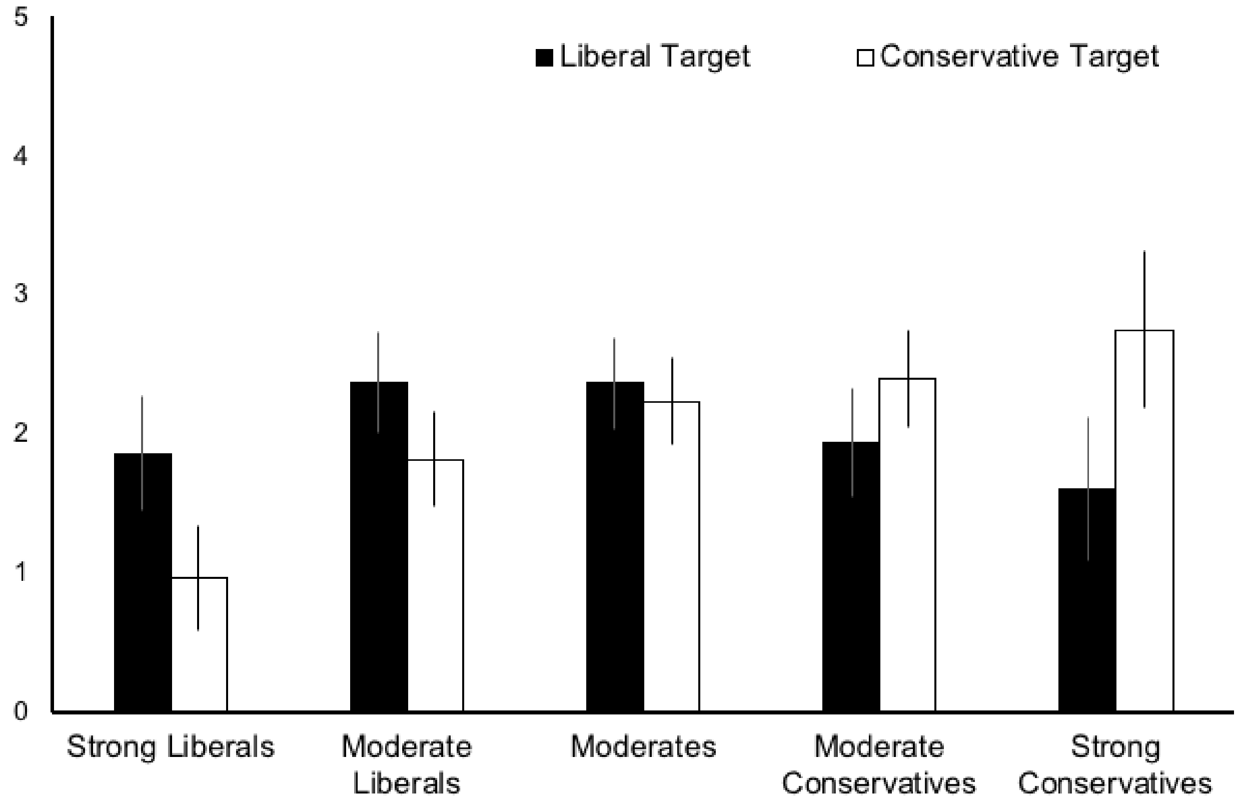

Figure SM1.5: *The Regression Intercepts and 95 % Confidence Intervals for Endorsement of the Sanctity/Degradation Foundation Depending on Experimental Condition, Ideological Type, and Ideological Extremity in Study 1*

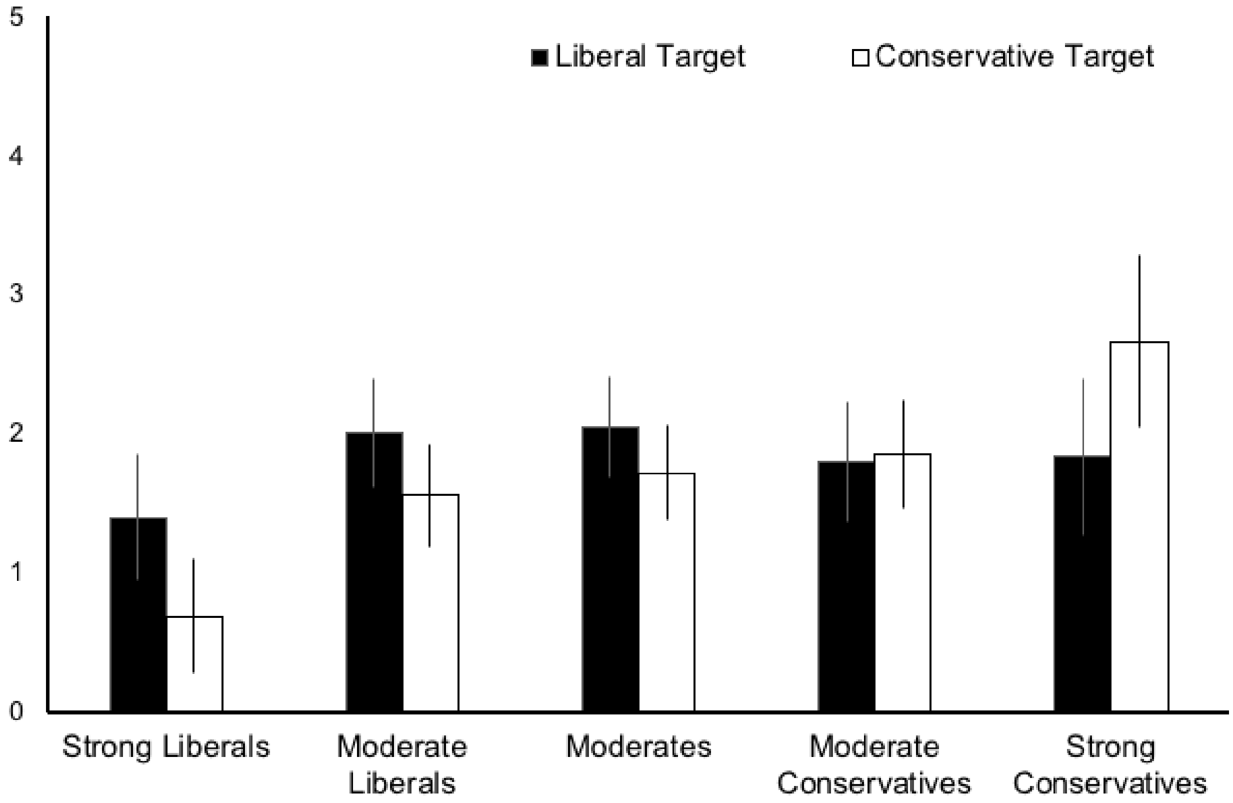

Figure SM2.1: *The Regression Intercepts and 95 % Confidence Intervals for Endorsement of the Care/Harm Foundation Depending on Experimental Condition, Ideological Type, and Ideological Extremity in Study 2*

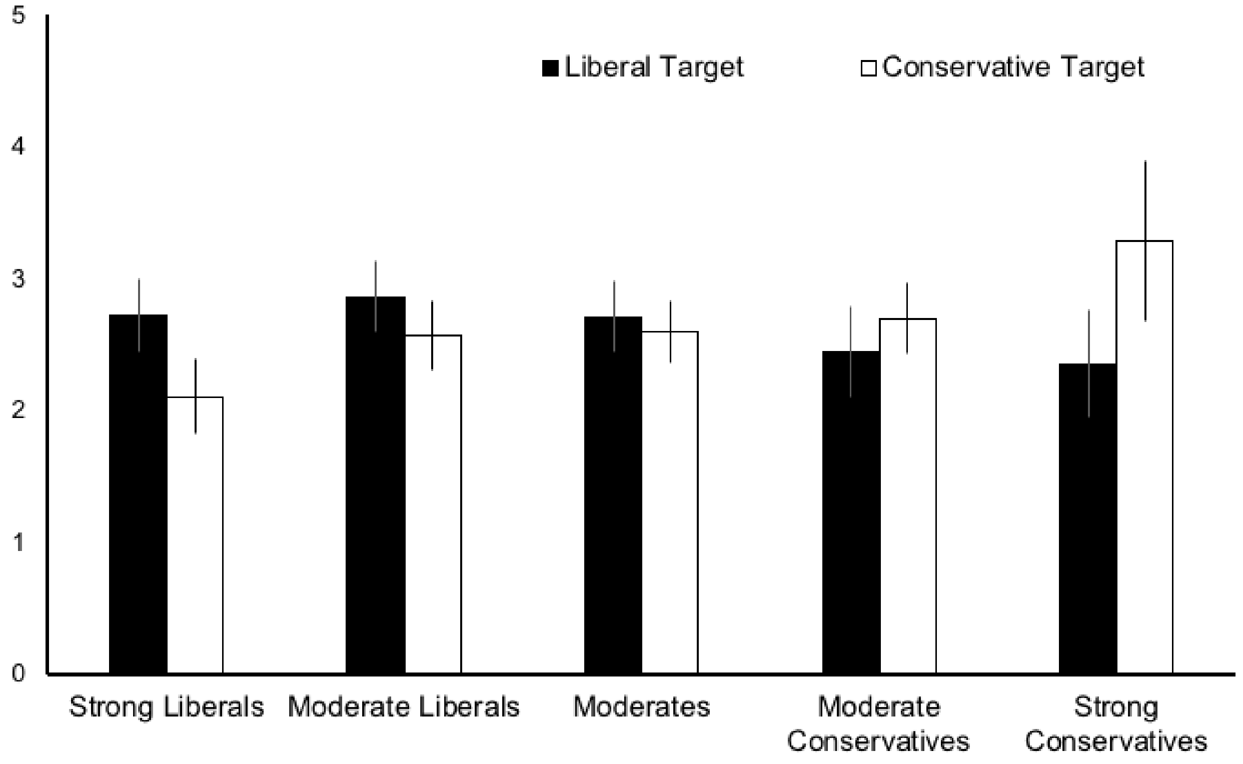

Figure SM2.2: *The Regression Intercepts and 95 % Confidence Intervals for Endorsement of the Fairness/Cheating Foundation Depending on Experimental Condition, Ideological Type, and Ideological Extremity in Study 2*

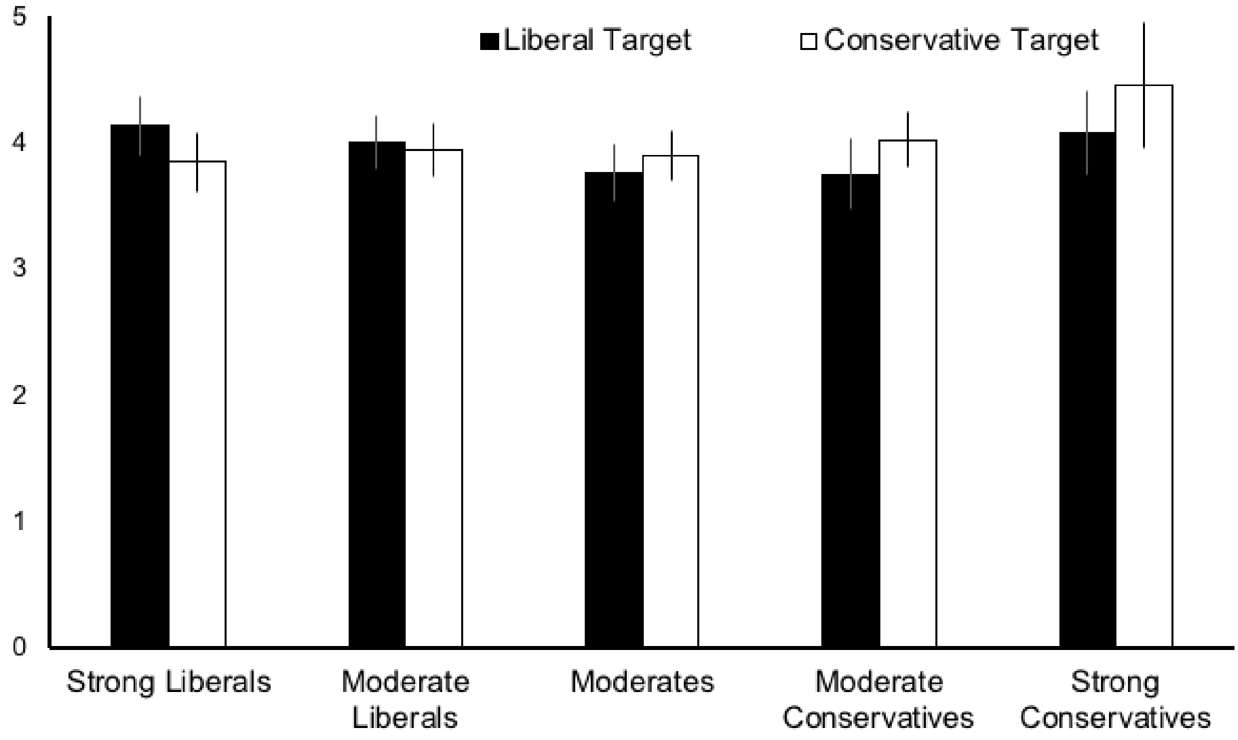

Figure SM2.3: *The Regression Intercepts and 95 % Confidence Intervals for Endorsement of the Loyalty/Betrayal Foundation Depending on Experimental Condition, Ideological Type, and Ideological Extremity in Study 2*

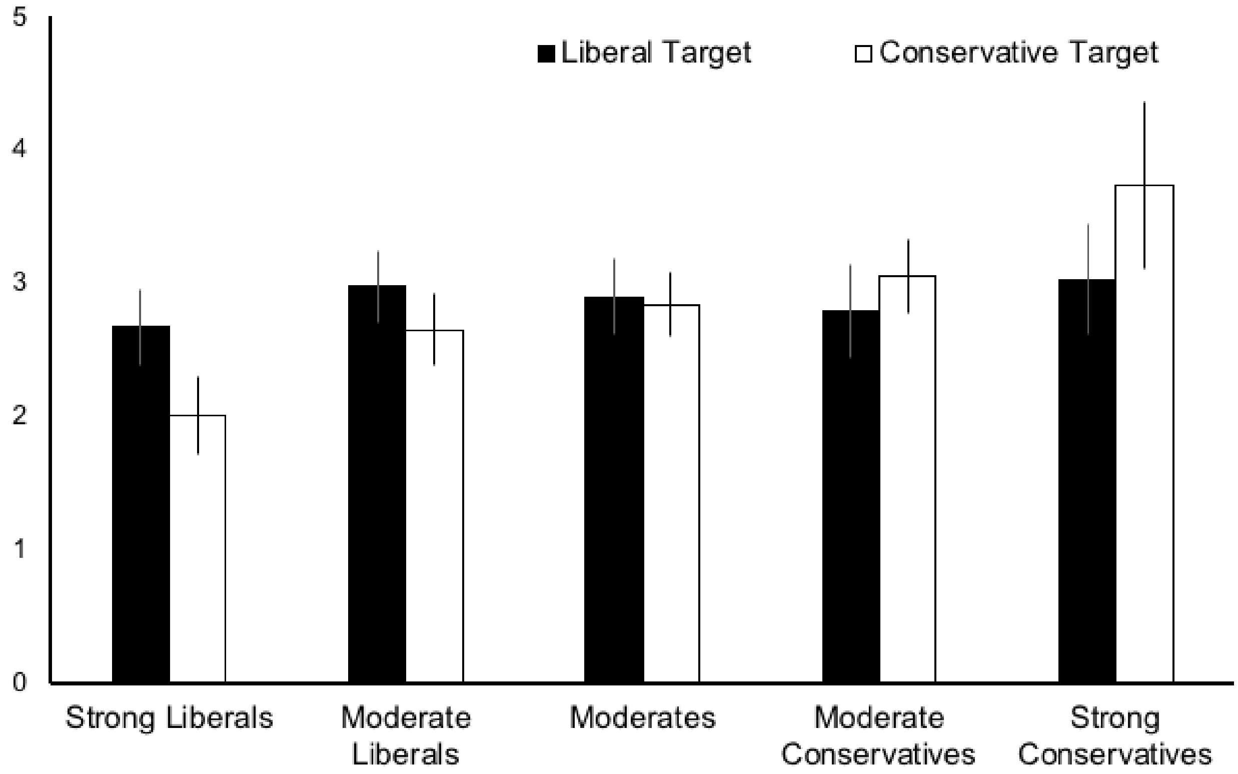

Figure SM2.4: *The Regression Intercepts and 95 % Confidence Intervals for Endorsement of the Authority/Subversion Foundation Depending on Experimental Condition, Ideological Type, and Ideological Extremity in Study 2*

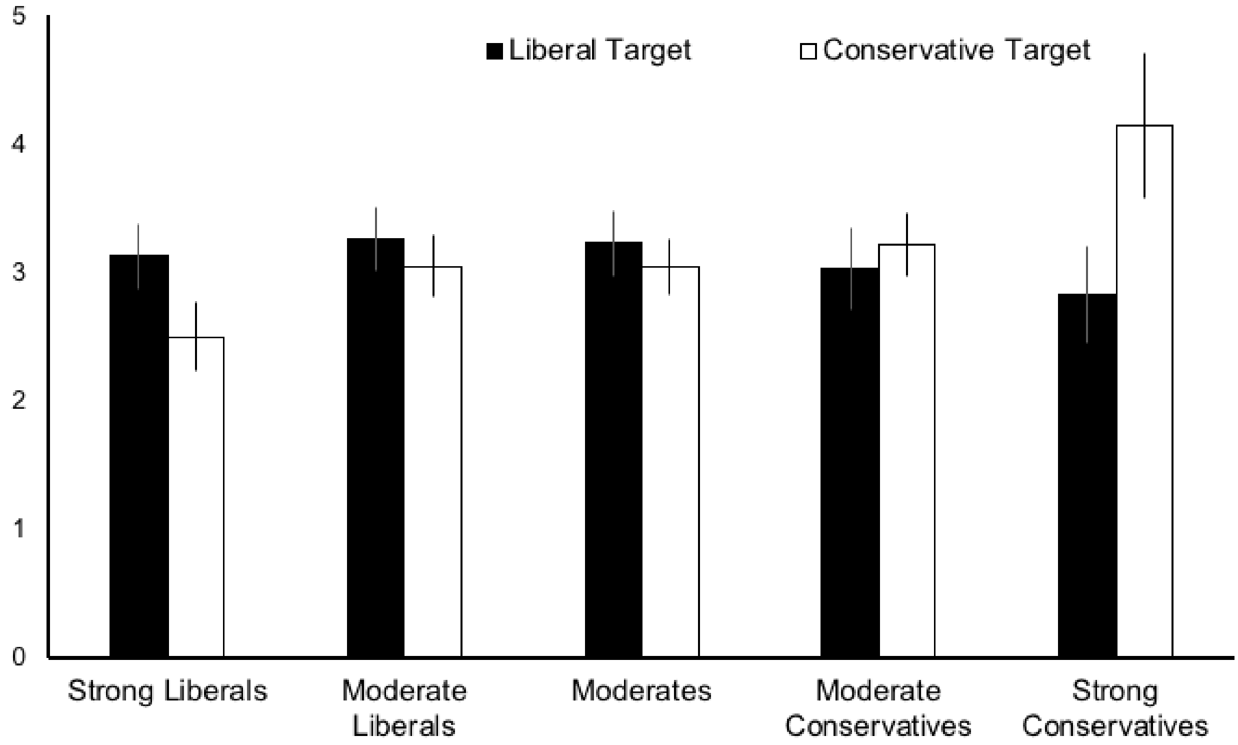

Figure SM2.5: *The Regression Intercepts and 95 % Confidence Intervals for Endorsement of the Sanctity/Degradation Foundation Depending on Experimental Condition, Ideological Type, and Ideological Extremity in Study 2*

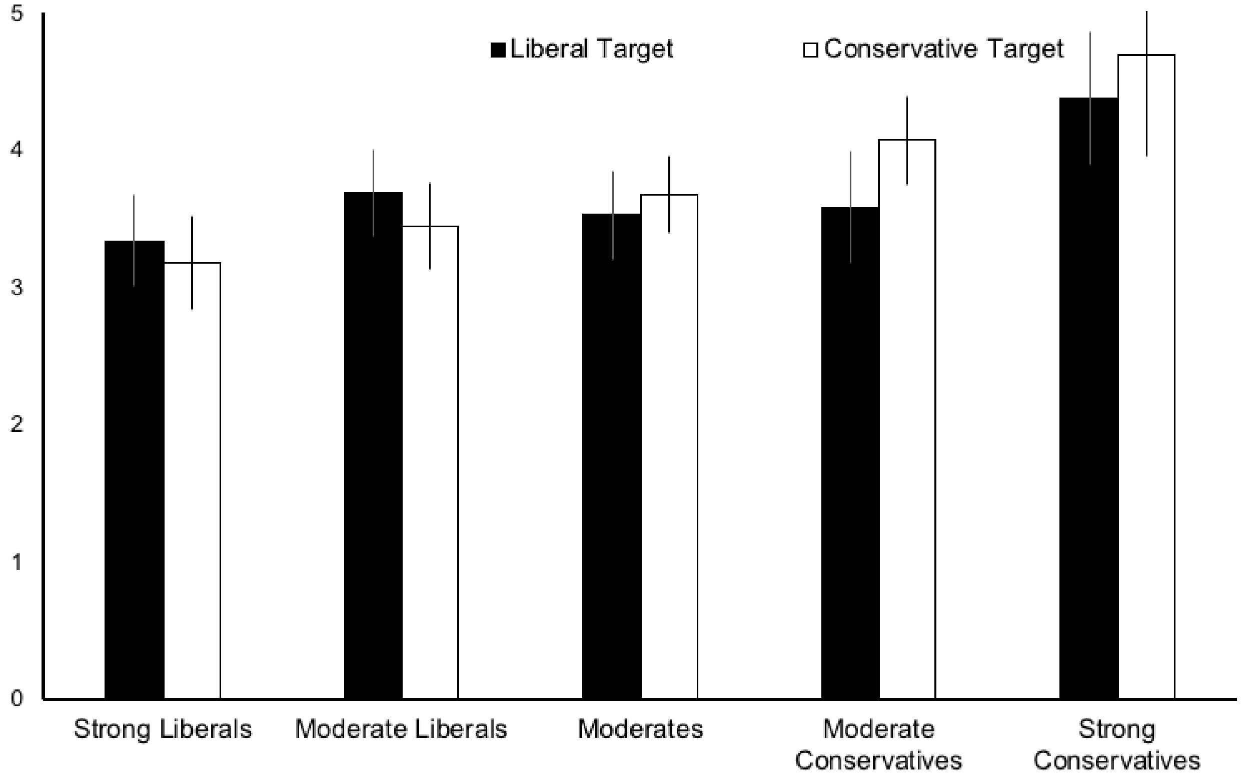

References

Feinberg, M., & Willer, R. (2015). From gulf to bridge: When do moral arguments facilitate political influence? *Personality and Social Psychology Bulletin, 41*(12), 1665-1681. https://doi.org/10.1177%2F0146167215607842

Frimer, J. A., Gaucher, D., & Schaefer, N. K. (2014). Political conservatives' affinity for obedience to authority is loyal, not blind. *Personality and Social Psychology Bulletin, 40*(9), 1205-1214. https://doi.org/10.1177%2F0146167214538672

Koch, A., Imhoff, R., Dotsch, R., Unkelbach, C., & Alves, H. (2016). The ABC of stereotypes about groups: Agency/socioeconomic success, conservative-progressive beliefs, and communion. *Journal of Personality and Social Psychology, 110*(5), 675-709. https://doi.org/10.1037/pspa0000046

Pew Research Center (2014). *Religious landscape study – political ideology*. Retrieved from http://www.pewforum.org/religious-landscape-study/political-ideology/

Schatz, R. T., Staub, E., & Lavine, H. (1999). On the varieties of national attachment: Blind versus constructive patriotism. *Political Psychology, 20*(1), 151-174. https://doi.org/10.1111/0162-895X.00140
